# Ancient viral integrations in marsupials: a potential antiviral defence

**DOI:** 10.1093/ve/veab076

**Published:** 2021-09-02

**Authors:** Emma F Harding, Alice G Russo, Grace J H Yan, Paul D Waters, Peter A White

**Affiliations:** School of Biotechnology and Biomolecular Sciences, University of New South Wales, UNSW Sydney, Sydney, NSW 2052, Australia; School of Biotechnology and Biomolecular Sciences, University of New South Wales, UNSW Sydney, Sydney, NSW 2052, Australia; School of Biotechnology and Biomolecular Sciences, University of New South Wales, UNSW Sydney, Sydney, NSW 2052, Australia; School of Biotechnology and Biomolecular Sciences, University of New South Wales, UNSW Sydney, Sydney, NSW 2052, Australia; School of Biotechnology and Biomolecular Sciences, University of New South Wales, UNSW Sydney, Sydney, NSW 2052, Australia

**Keywords:** endogenous viral element, marsupial, virus evolution, RNAi, piRNA, small RNA, paleovirology

## Abstract

Marsupial viruses are understudied compared to their eutherian mammal counterparts, although they may pose severe threats to vulnerable marsupial populations. Genomic viral integrations, termed ‘endogenous viral elements’ (EVEs), could protect the host from infection. It is widely known past viral infections and EVEs play an active role in antiviral defence in invertebrates and plants. This study aimed to characterise actively transcribed EVEs in Australian marsupial species, because they may play an integral role in cellular defence against viruses. This study screened publicly available RNA sequencing data sets (*n* = 35) and characterised 200 viral transcripts from thirteen Australian marsupial species. Of the 200 transcripts, 188 originated from either *Bornaviridae, Filoviridae*, or *Parvoviridae* EVEs. The other twelve transcripts were from putative active infections from members of the *Herpesviridae* and *Anelloviridae*, and *Hepadnaviridae*. EVE transcripts (*n* = 188) were mapped to marsupial genomes (where available, *n* = 5/13) to identify the genomic insertion sites. Of the 188 transcripts, 117 mapped to 39 EVEs within the koala, bare-nosed wombat, tammar wallaby, brushtail possum, and Tasmanian devil genomes. The remaining eight animals had no available genome (transcripts *n* = 71). Every marsupial has *Bornaviridae, Filoviridae*, and *Parvoviridae* EVEs, a trend widely observed in eutherian mammals. Whilst eutherian bornavirus EVEs are predominantly nucleoprotein-derived, marsupial bornavirus EVEs demonstrate a surprising replicase gene bias. We predicted these widely distributed EVEs were conserved within marsupials from ancient germline integrations, as many were over 65 million years old. One bornavirus replicase EVE, present in six marsupial genomes, was estimated to be 160 million years old, predating the American–Australian marsupial split. We considered transcription of these EVEs through small non-coding RNA as an ancient viral defence. Consistent with this, in koala small RNA sequence data sets, we detected *Bornaviridae* replicase and *Filoviridae* nucleoprotein produced small RNA. These were enriched in testis tissue, suggesting they could protect marsupials from vertically transmitted viral integrations.

## 1. Introduction

Australia’s extended geographic isolation has led to the evolution of varied and unique fauna, of which marsupials (Metatheria) are iconic members. Extant marsupials, that exist today only in Australia and America, are thought to have diverged from the eutherian lineage around 159 million years ago (Kumar et al. 2017). Due to this long evolutionary history, they retain unique reproductive traits that distinguish them from other mammals. A combination of factors including climate change and habitat destruction have pushed populations of many Australian marsupial species into decline, with over 100 of the >250 known species classified as endangered or vulnerable (EPBC 2020) for the EPBC Act list of threatened fauna.

Infectious diseases are a major threat to vulnerable species worldwide, including marsupials (Wayne et al. 2015). Some marsupial pathogens, such as chlamydia in koalas and mange (a parasitic mite) in wombats, have been well documented (Fraser et al. 2019), but very little is known about the diversity of viruses that infect Australian marsupials. Rapid viral mutation and recombination rates mean that the viruses can quickly decimate vulnerable populations when they arise, making their characterisation among marsupials essential (Hooper et al. 1999; Zhang et al. 2018). Prior studies have revealed that marsupial-infecting viruses encompass only a handful of the known viral families, including the *Poxviridae, Herpesviridae, Papillomaviridae, Picornaviridae*, and *Retroviridae* (Bennett et al. 2010; Amery-Gale et al. 2014; Carneiro et al. 2018; Harvey et al. 2019). However, the discovery of these viruses has largely been contingent on severe and noticeable disease symptoms, including lesions, behavioural changes, and blindness (Hooper et al. 1999; Dunowska et al. 2012; Sarker et al. 2017). The lack of knowledge of viruses that affect Australian marsupials makes it difficult to assess the threat that viral infection poses to declining populations.

During a viral infection, there is a chance that viral DNA or complementary DNA will integrate into the host genome in a process called endogenisation, creating endogenous viral elements (EVEs) that can be passed on to progeny (Holmes 2011). Paleovirology—the study of EVEs—has rapidly exposed the ubiquity of EVEs throughout vertebrate and invertebrate genomes (Kryukov et al. 2019; Kawasaki et al. 2021). Due to the obligate integration of retroviral genomes into the host chromosome during replication, most mammalian EVEs originate from ancient retroviruses; however non-retroviral EVEs exist widely, particularly in invertebrates, and are thought to integrate with the assistance of reverse transcriptases encoded by host retroelements, e.g. long interspersed nuclear elements (Horie et al. 2010).

Non-retroviral EVEs have been identified in vertebrate genomes from a variety of viral families, including the *Bornaviridae, Filoviridae*, *Parvoviridae*, *Circoviridae*, and *Hepadnaviridae* (Belyi, Levine, and Skalka 2010a,b; Horie et al. 2010; Katzourakis and Gifford 2010; Liu et al. 2011). Of these, EVEs from the *Bornaviridae*, *Filoviridae*, and *Parvoviridae* are known to be present in three marsupial genomes (Belyi, Levine, and Skalka 2010b; Smith et al. 2016). However, due to a lack of genomic data, EVEs have been identified in only a handful of species, including the tammar wallaby, Tasmanian devil, and koala (Kawasaki et al. 2021). No annotation of the EVEs identified in marsupials has thus far taken place, and viral gene integration bias has not been investigated (Kapoor, Simmonds, and Lipkin 2010; Taylor, Leach, and Bruenn 2010).

Whilst EVEs were originally thought to serve no function, as they usually accumulate mutations over millions of years and the majority no longer express functional proteins, a cellular role for some EVE derived RNA or proteins has been identified (Honda and Tomonaga 2016). For example, EVEs that integrate and remain largely intact in the genome can be transcribed and sometimes translated into viral proteins using host machinery (Fujino et al. 2014). These proteins have been implicated in cellular antiviral defences; EVE-encoded proteins can act as restriction factors against infection from related viruses by acting as host cell receptor antagonists to limit viral entry or interfering with viral polymerase activity (Fujino et al. 2014; Frank and Feschotte 2017; Kim et al. 2020). EVE-encoded messenger RNA (mRNA) can also play an antiviral role as non-coding RNA (ncRNA). One well-studied class of ncRNA is small interfering RNA (siRNA), a molecule that is primarily involved in embryonic development in mammals but which also serves as a primary immune defence against viruses in invertebrates (Blair 2011; Tassetto et al. 2019). The identification of EVEs and analysis of transcription patterns can not only inform about virus–host coevolution but may also hint at a transcriptional, regulatory, antiviral or protective function of these sequences in both marsupials and eutherian mammals.

In this study, we screen publicly available RNA-sequencing (RNA-Seq) data to identify actively transcribed EVEs in Australian marsupials. We identify an abundance of nucleoprotein and replicase EVE-derived transcripts across thirteen Australian marsupial species from the *Bornaviridae* and *Filoviridae* families. We show that for the koala, *Bornaviridae* replicase and *Filoviridae* nucleoprotein EVEs are processed into small RNA, leading to the intriguing possibility of an RNA defence system.

## 2. Materials and methods

### 2.1 Selection of publicly available RNA-seq data sets to screen for viral sequences

Raw RNA-Seq read collections (*n* = 35) were retrieved for 13 animals from the NCBI Sequence Read Archive (SRA) using keyword searches of marsupial genera and common names. Marsupial species were selected based on RNA-Seq data availability: four data sets each from the Tasmanian devil (*Sarcophilus harrisii*), the tammar wallaby (*Notamacropus eugenii*), the long-nosed bandicoot (*Perameles nasuta*), the fat-tailed dunnart (*Sminthopsis crassicaudata*), the bare-nosed wombat (*Vombatus ursinus*), the koala (*Phascolarctos cinereus*), and the sugar glider (*Petaurus breviceps*). One data set was downloaded for the false antechinus (*Pseudantechinus macdonnellensis*), Southern brown bandicoot (*Isoodon obesulus*), striped possum (*Dactylopsila trivirgata*), Western pygmy possum (*Cercartetus concinnus*), brushtail possum (*Trichosurus vulpecula*), and yellow-footed rock wallaby (*Petrogale xanthopus*) (Supplementary Table S1). All raw reads were subject to FastQC (version 0.11.8, (Andrews 2010)) analysis prior to assembly.

### 2.2 Identification of transcribed EVE sequences

**Figure 1.**
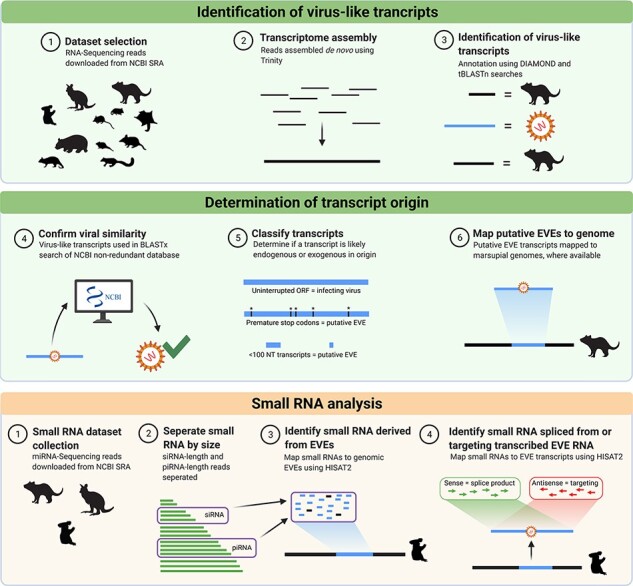
The bioinformatics workflow used in this study to identify viral transcripts from thirty-five marsupial RNA-Seq data sets. Publicly available data sets were downloaded from NCBI SRA and quality checked using FastQC before assembly into transcriptomes, using Trinity. DIAMOND was used to annotate each assembled transcript as host, viral, or other. Viral transcripts were filtered to merge overlapping hits and remove duplicate hits. To confirm the viral origin of each transcript, a reciprocal BLASTx search of the NCBI nr protein database was performed. Transcripts were classified as EVEs if they mapped to the representative marsupial genome (where available), or putative EVEs if they had interrupted reading frames, identity to confirmed EVEs, or were from frequently endogenised viral families. Small RNA analysis was undertaken to identify if any EVEs gave rise to small RNA molecules. Figure created using BioRender.com.

Raw read data sets (*n* = 35) were quality trimmed using Trimmomatic (version 0.38; Bolger, Lohse, and Usadel 2014) and assembled *de novo* using Trinity (v.2.5.1, Grabherr et al. 2011) (Fig. 1, step 2).

To identify EVE trancripts present within each data set, the assembled contigs were annotated using a BLASTx search of the non-redundant protein database using DIAMOND (v0.9.10) (Fig. 1, step 3) (Buchfink, Xie, and Huson 2015). The E-value cut-off for BLASTx annotation was set to 1e^−03^ to detect divergent sequences, and all other settings were left as default.

As DIAMOND is an optimised BLAST algorithm designed to rapidly find hits in large data sets and fail to annotate some viral transcripts, transcriptomes were then screened manually to capture the full diversity of viral transcripts. Using custom sets of proteins compiled from the NCBI Reference Sequence database (RefSeq), a tBLASTn search (default BLOSUM62 scoring matrix, E-value cut-off of 1e^−03^) was conducted against each marsupial transcriptome to identify additional viral transcripts (Fig. 1, step 3). A custom Python (v3.7.4) script was written to consolidate the tBLASTn results from the screening, identifying duplicate hits, and merging high-scoring pairs (HSPs; alignments) that were within 100-nucleotide (nt) vicinity of other HSPs.

To eliminate false positives (transcripts with viral homology when translated but that did not originate from a viral sequence), each sequence was compared to the NCBI nr protein/nt database using a reciprocal BLASTx search with an E-value cut-off of 1e^−03^ to confirm identity to viral proteins (Fig. 1, step 4). Any sequence that, when translated, did not have similarity to a viral protein was considered a false hit and disregarded. The top hit was used to classify each sequence. All statistics about the sequences obtained were based on the top HSP result returned by the BLASTx search.

Transcribed EVE sequences were identified using the following criteria: firstly, transcripts were mapped back to their representative marsupial genome (where available, *n* = 5) using a BLASTn search (default settings, E-value 1e^−03^). They were confirmed as an EVE if they mapped identically to genomic DNA (Fig. 1, step 6; Supplementary Table S2). Five of the thirteen marsupial species in this study have published genomes the Tasmanian Devil (mSarHar1.11) (Miller et al. 2011), koala (phaCin_unsw_v4.1) (Johnson et al. 2018), tammar wallaby (Meug_1.1) (Renfree et al. 2011), common wombat (bare-nosed wombat genome assembly) (Janke et al. 2002; Nilsson et al. 2003), and brushtail possum (VGP: 2020) for the Vertebrate Genomes Project citation.

If no genome was available for the animal, we then used five further criteria to determine the likelihood of the transcript to be endogenous in origin and therefore a putative EVE (Fig. 1, step 5; Supplementary Table S2). Firstly, each transcript was *in silico* translated and inspected for premature stop codons. To account for possible sequencing or assembly errors, transcripts needed to contain multiple (>2) premature stop codons before they were classified as putative EVEs. Secondly, the identity of the transcript to previously characterised and confirmed mammalian EVEs was assessed, including EVEs confirmed in this study (>97 per cent AA identity to known EVE). Transcripts <300 nt in length with identity to a frequently endogenised family (*Bornaviridae*, *Filoviridae*, or *Parvoviridae*) were also classified as putative EVEs. Relaxed criteria were used to account for the expected divergence of marsupial EVEs from known viral sequences.

If the transcript did not fit any of the above criteria, it was classified as a putative infecting virus.

### 2.3 Analysis of small-RNA data sets

As the EVEs identified in this study were unlikely to be translated into functional proteins, the ability for them to be spliced into small RNA was investigated. Small RNA sequencing data sets were retrieved from the NCBI Sequence Read Archive and then quality tested and trimmed with TrimGalore! (Galaxy Version 0.63) on the Galaxy Australia server (Afgan et al. 2018). Reads were separated into siRNA and piRNA based on length (20–22 nt and 23–29 nt, respectively) and mapped to marsupial EVE sequences (transcripts and genomic EVEs) using HISAT2 (Galaxy Version 2.1.0 + galaxy5) (Kim, Langmead, and Salzberg 2015; Afgan et al. 2018) with default settings. The resulting aligned reads were extracted and mapped back to EVE sequences in Geneious with the Geneious map to reference tool, with no mismatches tolerated.

### 2.4 Estimating integration time of transcriptionally active EVEs

The integration times of transcribed EVEs were estimated by identifying orthologous sequences across species using a BLASTn search of marsupial genomes in Ensembl (Yates et al. 2020). Searches were conducted against genomes available on Ensembl, the tammar wallaby (Meug_1.0) (Renfree et al. 2011), koala (phaCin_unsw_v4.1) (Johnson et al. 2018), Tasmanian devil (DEVIL7.0) (Murchison et al. 2012), and wombat (bare-nosed_wombat_genome_assembly) (Janke et al. 2002; Nilsson et al. 2003) genomes. The brushtail possum genome was not available on Ensembl (obtained from the VGP: Vertebrate Genomes Project 2020), so separate BLASTn searches were conducted for this genome using Geneious Prime. For integrations with >80 per cent similarity between species, orthology was tested using a BLASTn search of the eukaryotic flanking sequences on Ensembl. Integrations with significant BLASTn hits (E-value < 1e^−03^, nt identity <85 per cent) to the genomic region 500 bp upstream of the EVE were considered orthologous. An EVE was considered widely conserved if it was present in three or more distinct marsupial species.

To better estimate the integration time of each EVE, they were also searched for in an American marsupial (short-tailed opossum, *Monodelphis domestica*) (ASM229v1) (Mikkelsen et al. 2007), a basal eutherian (nine-banded armadillo, *Dasypus* novemcinctus) (Dasnov3.0) (unpublished), and a monotreme (platypus, *Ornithorhynchus anatinus*) (mOrnAna1.p.v1) (VGP: Vertebrate Genomes Project 2020) genome. To complement this, a BLASTn search of transcribed EVEs in Geneious was undertaken to identify similar transcripts in the eight marsupials from this study with no published genome. Transcripts with significant hits (E-value < 1e^−03^, nt identity > 85 per cent) were classified as similar.

The integration time of each EVE was estimated from the marsupial evolutionary tree, generated by TimeTree (Kumar et al. 2017). The presence or absence of EVEs and flanking regions in each marsupial species was used to estimate the time of viral to host integration (Katzourakis and Gifford 2010). EVEs that were present in two species but not a third integrated after the divergence of the third species but before the divergence of the first two.

## 3. Results

### 3.1 Diversity of viral transcripts present in Australian marsupials

**Figure 2.**
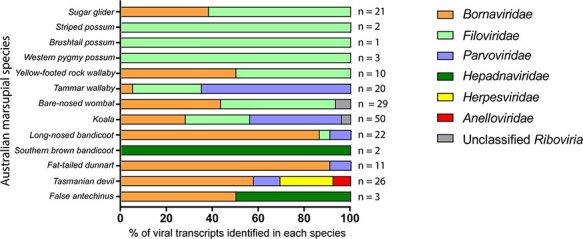
Classification of viral-like transcripts present in Australian marsupial data sets. Thirty five RNA-Seq data sets from thirteen Australian marsupial species were screened for the presence of viral-like transcripts using a genome-independent bioinformatics workflow. Viral-like transcripts were grouped into families based on the top hit of the reciprocal BLASTx nr search. The colours represent the viral family of each transcript top BLASTx hit indicated in the figure legend. The total number of viral-like transcripts identified in each species is shown on the right of the bar graph.

Marsupial RNA-Seq data sets were screened for the presence of viral transcripts to determine the diversity of viral families represented. A total of 200 viral transcripts were identified in 13 Australian marsupial species, from 35 RNA-Seq data sets (Fig. 2). All sequences, when translated *in silico*, exhibited similarity to proteins from one of six viral families: *Bornaviridae* (*n* = 85, 42 per cent), *Filoviridae* (*n* = 60, 30 per cent), *Parvoviridae* (*n* = 39, 20 per cent), *Herpesviridae* (*n* = 6, 3 per cent), *Hepadnaviridae* (*n* = 4, 2 per cent), and *Anelloviridae* (*n* = 2, 1 per cent), with the remaining four transcripts unclassifiable beyond the *Riboviria* realm (Fig. 2).

### 3.2 Filoviridae, Bornaviridae, *and* Parvoviridae *sequences are widely endogenised*

**Figure 3.**
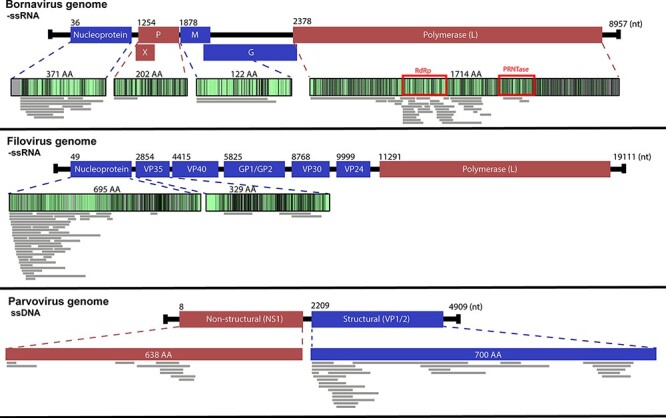
Alignment of endogenous viral element (EVE) transcripts with similarity to Bornaviridae, Filoviridae, and Parvoviridae sequences. Sequences were identified from marsupial RNA-Seq reads using a bioinformatics workflow and the viral identity was determined with a BLASTx search against the NCBI non-redundant database. Sequences were translated and mapped to the viral protein with which they share the highest identity. Grey lines represent transcripts from the thirty-five marsupial transcriptomes. Genes coloured blue are structural genes, and genes coloured red are non-structural genes. The proteins are gradated based on the conservation of amino acids between members of the viral families; light green represents conserved regions and grey represents more variable regions. Red boxes highlight areas containing recognised viral enzymatic motifs.

Viral-like sequences can either be derived from the host genome or from an infecting extant virus. Our analysis identified 200 viral transcripts, of which 188 transcripts had identity to genes from the *Bornaviridae*, *Filoviridae*, and *Parvoviridae* families, as well as some unclassifiable viruses below the realm *Riboviria* (Figs 2, 3; Supplementary Table S2). Of these transcripts, 117 were mapped to marsupial genomes, with the other 71 transcripts were from marsupials with no available genome. The mapped transcripts were derived from 39 unique genomic locations in the koala (*n* = 12/39), bare-nosed wombat (*n* = 6/39), tammar wallaby (*n* = 13/39), brushtail possum (*n* = 1/39), and Tasmanian devil (*n* = 7/39) (Supplementary Table S3).

The *Herpesviridae* and *Anelloviridae*-like transcripts in the Tasmanian devil did not map to the genome and contained no stop codons and therefore were from putative active infections. The *Herpesviridae*-like transcripts were all identified from peripheral blood mononuclear cells and when translated *in silico* had identity to the ORF57 transcriptional control protein and ORF58 proteins of Bovine Gammaherpesvirus 6, two proteins with no eukaryotic homologues. The *Anelloviridae*-like transcripts were also identified from peripheral blood mononuclear cells and when translated *in silico* had identity to Torque Teno 2 *Leptonychotes weddellii* (Weddell seal) virus 2 (NC_035135). Four *Hepadnaviridae*-like transcripts were identified in the false antechinus and the Southern brown bandicoot and could not be accurately classified.

### 3.3 Viral replicase and nucleoprotein genes are over-represented in EVE transcripts

**Figure 4.**
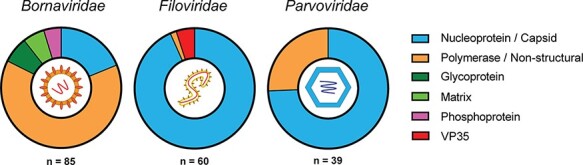
Viral gene of origin for EVEs identified in this study. EVE transcripts were identified using a bioinformatics pipeline and classified into family and gene of origin based on the top hit in a BLASTx search of the NCBI non-redundant database. Colours represent the different gene categories and are detailed in the figure legend. Viral icons depicting the basic viral structure for each family are shown in the centre of the pie chart. The number of EVE transcripts that mapped to each family are shown below the pie chart.

Transcripts were identified from marsupial RNA-Seq (*n* = 188), with identity to commonly endogenised viral families. Viral gene bias in these transcripts was investigated to compare with reported trends seen in eutherian mammals. The most numerous transcripts mapped to negative ssRNA (−ssRNA) viruses (145/188 transcripts) (Fig. 3; Supplementary Table S2). Of these, *Bornaviridae*-like transcripts were the most ubiquitous (*n* = 85/188, 45 per cent), present in 9/13 marsupial species (Fig. 2; Supplementary Table S2). These transcripts mapped to the RNA-dependent RNA polymerase domain of the L (large) protein (*n* = 54/85), nucleoprotein (*n* = 16/85), glycoprotein (*n* = 6/85), matrix protein (*n* = 5/85), or phosphoprotein (*n* = 4/85) (Figs 3, 4; Supplementary Table S2).

EVE transcripts that mapped to the −ssRNA *Filoviridae* genes were also abundant, present in 9/13 species, with a total of 60/188 (32 per cent) sequences identified (Fig. 2). *Filoviridae*-like transcripts were identified in the wombat, koala, long-nosed bandicoot, sugar glider, tammar wallaby, striped possum, Western pygmy possum, brushtail possum, and yellow-footed rock wallaby species (Fig. 2; Supplementary Table S2). These transcripts, when translated *in silico*, were all similar to human filovirus proteins and mapped to the nucleocapsid (*n* = 56/60), VP35 (*n* = 3/60), and L (*n* = 1/60) genes (Figs 3, 4; Supplementary Table S2).

In addition to −ssRNA viruses, numerous transcripts mapped to ssDNA *Parvoviridae* family genes (*n* = 39/188) (Supplementary Table S2). *Parvoviridae*-like transcripts were common throughout the data sets, appearing in five of the thirteen marsupial species studied: the fat-tailed dunnart, the koala, the long-nosed bandicoot, the tammar wallaby, and the Tasmanian devil (Fig. 2). Marsupial transcripts mapped to either the structural capsid genes *VP1/VP2* (*n* = 28/39) or the non-structural genes (*n* = 11/39) (Figs 3, 4; Supplementary Table S2). The top BLASTx results for these transcripts were primarily viral sequences from eutherian mammals (*n* = 35/39); however, some were from avian (*n* = 3/39) and reptilian (*n* = 1/39) parvoviruses (Supplementary Table S2).

Some transcripts from the koala and wombat had BLASTx hits to viral proteins from the *Riboviria* realm and could not be further classified with this methodology (Fig. 2). These transcripts included three putative *nucleoprotein* encoding sequences with identity to sequences from Guangdong red-banded snake chuvirus-like virus (*n* = 3; 44.2 per cent identity over 74 aa, 42.9 per cent identity over 62 aa, 36.4 per cent over 76 aa, GenPept: AVM87274), as well as one hypothetical protein encoding sequence similar to Sanxia atyid shrimp virus 4 (*n* = 1; 45.5 per cent identity over 43 aa, GenPept: YP_009337430).

### 3.4 Bornavirus replicase and filovirus nucleoprotein EVE sequences are represented as small RNA in koala testis

In the present study, we describe EVEs that are transcriptionally active throughout Australian marsupial species. To determine if any of these EVEs give rise to small RNA sequences, including siRNA or PIWI protein-interacting RNA (piRNA), we analysed thirty-five available marsupial small RNA data sets from the koala (*n* = 10), Tasmanian devil (*n* = 15), and tammar wallaby (*n* = 10) (Supplementary Table S4). piRNA traditionally targets transposons and rises either from genomic insertions or is spliced from mRNA transcripts. siRNA is involved in antiviral defence in invertebrates and targets incoming RNA for degradation. No small RNA sequences from the Tasmanian devil or tammar wallaby data sets mapped to EVEs identified in this study, likely due to the significantly lower read depth of these data sets (Supplementary Table S4). Due to this, no small RNA analysis was conducted on these animals.

All ten of the koala small RNA data sets contained reads that mapped to EVE transcripts and genomic integrations. Of the twelve transcriptionally active koala EVEs identified in this study, 99 per cent mapped to bornavirus *replicase* and filovirus *nucleoprotein* EVEs (Supplementary Table S5). The bornavirus nucleoprotein (pcEBLN), parvovirus capsid (pcEPLVP) or chuvirus-like nucleoprotein (pcECLNP) koala EVEs were disregarded from further analysis due to few small RNA molecules mapped.

**Figure 5.**
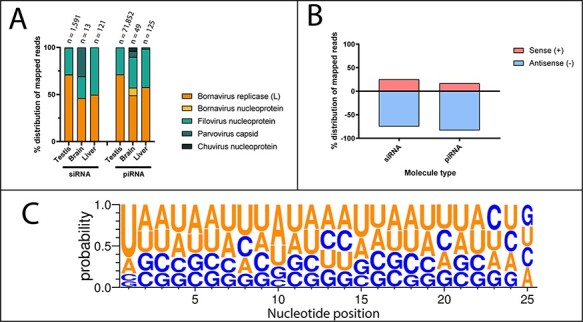
Analysis of small RNA reads that map to viral integrations in the koala genome. Small RNA data sets were downloaded from NCBI SRA and mapped to EVEs in the koala genome identified in this study using HISAT2. (A) Distribution of mapped small RNA between genomic EVEs in the koala, grouped by tissue type. Viral family and genes are coloured and detailed in the key. (B) Orientation of small RNA mapped to EVE-derived transcripts in the koala. (C) Sequence logo of secondary piRNA-length molecules, showing 1ʹU bias and 10ʹA bias. Created using WebLogo 3 (Crooks et al. 2004).

The bornavirus *replicase* and filovirus *nucleoprotein* EVEs gave rise to primarily unidirectional small RNA molecules with a 1ʹU bias characteristic of primary piRNA (Fig. 5, panel C). A smaller subset (0.03–1.92 per cent) of mapped small RNA molecules had a 10ʹA bias, a marker of ping-pong piRNA amplification (Fig. 5, panel C). Of the small RNA that mapped to EVE-derived transcripts, 83.3 per cent of the piRNA-length reads and 74.8 per cent of the siRNA-length reads were antisense to the transcripts, suggesting they bind target EVE transcripts. piRNA is primarily utilised in germline cells and reproductive tissues to prevent transposon-induced chromosomal damage. In the koala, we compared EVE-derived small RNA molecules in testis and somatic cells. Testis samples had the highest number of small RNA mapping to genomic EVEs: between 0.35 per cent and 0.47 per cent of all cellular piRNA-length molecules and between 0.07 per cent and 0.35 per cent of all siRNA-length molecules. These high levels of transcripts that mapped to EVEs were not seen in koala liver or brain samples where between 0 per cent and 0.1 per cent of small RNA molecules mapped to EVEs.

### 3.5 Marsupial EVEs have been conserved over millions of years of host evolution

We identified transcriptionally active EVEs from the *Bornaviridae, Filoviridae, Parvoviridae*, and *Chuviridae* families in thirteen Australian marsupials, some of which give rise to small RNA molecules. To determine if these active EVEs arose from multiple integration events or are from conserved ancient integrations, genomic searches were undertaken to identify orthologous EVEs in other marsupial and mammalian (armadillo and platypus) species.

**Figure 6.**
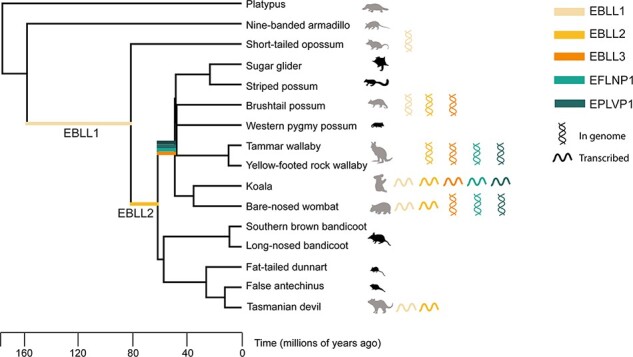
Estimated integration times of endogenous viral elements identified in this study. BLASTn searches of marsupial genomes using Ensembl and transcriptomes using Geneious Prime was conducted to identify EVEs present in Australian marsupials. EVEs present in three or more marsupial species were identified and their integration times estimated using an evolutionary tree of marsupials generated by TimeTree. The coloured lines on the tree represent estimated integration times with the colours corresponding to the different EVEs as depicted in the figure legend. Animals with available genomes are shaded grey. Double helices denote the presence of the EVE in the animal genome, and single lines represent active transcription of the EVE. The scale bar on the tree represents million years of evolution.

With the exception of EVEs shared between the closely related koala and wombat species, most were present in a single marsupial. However, two bornavirus replicase EVEs, one filovirus nucleoprotein EVE and one parvovirus capsid EVE, were present in three or more marsupial genomes (Fig. 6). The oldest integration identified was a bornavirus replicase EVE of 3050 nt, estimated to be up to 160 million years old and (Fig. 6) and named marsupial EBLL1. This EVE was present in the bare-nosed wombat, Tasmanian devil, brushtail possum, and opossum genomes, and smaller fragments could be detected in the wallaby (Fig. 6 and Supplementary Table S4). This integration was not identified in the platypus or armadillo genomes, placing the estimated integration time after the eutherian–marsupial split 160 million years ago but before the Australian–South American marsupial divergence 82 million years ago (Fig. 6).

The second most widespread EVE, a second bornavirus replicase EVE (560 nt), termed ‘marsupial EBLL2’, was estimated to be a successive integration some time between 62 and 82 million years ago. EBLL2 was detected in the genomes of the bare-nosed wombat, tammar wallaby, and Tasmanian devil but not in the opossum, armadillo, or platypus genomes (Fig. 6). This places the estimated integration time after the Australian–South American marsupial split but before the Australian marsupial radiation 62 million years ago (Fig. 6).

In contrast to the ancient bornavirus replicase integrations, filovirus nucleoprotein EVEs were estimated to be more recently integrated. One EFLNP integration was conserved in the koala, bare-nosed wombat, and tammar wallaby, placing the estimated integration time after the diprotodont radiation 49 million years ago but before the macropodiform split (Fig. 6).

Conserved parvovirus EVEs were also more recent integrations, estimated to be similar ages to the filovirus EVEs. One EPLVP was present in three marsupial species: the tammar wallaby, koala, and wombat, estimated to be up to 65 million years old (Fig. 6).

## 4. Discussion

Our study provides evidence that EVE integrations that encode bornavirus replicase or filovirus nucleoprotein genes are ubiquitous throughout marsupials and are actively transcribed into non-coding transcripts and small RNA. These integrations are conserved throughout marsupial evolution and date up to 182 million years ago. Consistent with the trends in eutherian mammals, they mapped only to the *Filoviridae*, *Bornaviridae*, and *Parvoviridae* families and *Ribovaria* realm in thirteen Australian marsupial species; however, there was a high prevalence of *Bornaviridae* replicase genes not seen in eutherian mammals (Kawasaki et al. 2021). EVEs from these families have only previously been identified in the tammar wallaby and Tasmanian devil genomes (Kapoor, Simmonds, and Lipkin 2010; Taylor, Leach, and Bruenn 2010). This study provides novel evidence that EVEs are transcriptionally active in a range of marsupial hosts and tissues, including the fat-tailed dunnart, long-nosed bandicoot, Southern brown bandicoot, striped possum, brushtail possum, Western pygmy possum, yellow-footed rock wallaby, and false antechinus (Fig. 2; Supplementary Table S2).

This workflow also identified four possible infecting viruses. A further twelve transcripts were identified that were sourced from a Gammeherpesvirus and Anellovirus in the Tasmanian devil and a Hepadnavirus in the Southern brown bandicoot and false antechinus. These putative active infections require further investigation.

### 4.1 Nucleoprotein and polymerase genes are overrepresented in transcribed EVEs

Over 40,000 viruses infect mammals; however, very few are endogenised (Carlson et al. 2019). Through continual screening of mammalian genomes, it is evident that certain viral families are consistently overrepresented as non-retroviral EVEs, namely *Bornaviridae, Parvoviridae*, and *Filoviridae* (Belyi, Levine, and Skalka 2010a,b). This repeated pattern of integration and conservation within genomes has been extensively observed throughout a range of vertebrate species in prior studies but has not been described in marsupials other than the few model species (Horie et al. 2010; Kawasaki et al. 2021). This study expands the observation to five Australian marsupial genomes and eight transcriptomes, in addition to the previously studied opossum. Furthermore, the current study proposes the origin of the *Bornaviridae* family to be a minimum of 80 million years old (Fig. 6), similar to estimates from a recent vertebrate-wide study (Kawasaki et al. 2021).

*Bornaviridae* and *Parvoviridae* viruses replicate in the nucleus, which may lead to more frequent genomic integration; however, *Filoviridae* viruses replicate in the cell cytoplasm and would not be expected to interact with DNA or reverse transcriptases regularly (Hume and Mühlberger 2019). Additionally, if replication site was a factor in the frequency of endogenisation, large double-stranded DNA viruses like *Herpesviridae* and RNA viruses like *Orthomyxoviridae* which also replicate in the nucleus should be endogenised at a similar rate (Morissette and Flamand 2010; Dou et al. 2018). The disproportional integration and transcription of *Bornaviridae*, *Filoviridae*, and *Parvoviridae* suggest an evolutionary basis that should be explored further.

Interestingly, whilst the most prevalent integrations in eutherian mammals are nucleocapsid genes from the *Bornaviridae* and *Filoviridae* families (Horie et al. 2010; Katzourakis and Gifford 2010; Belyi, Levine, and Skalka 2010b), in marsupials the bornavirus polymerase (L) gene is more commonly integrated and the eutherian nucleocapsid bias is not as prominent (Fig. 3). During a bornavirus infection, there is a transcription gradient from 3ʹ to 5ʹ, resulting in a much higher ratio of nucleoprotein mRNA compared to the other genes (Whelan, Barr, and Wertz 2004). If retrotransposon integration events are random, this transcription gradient will result in a higher rate of nucleoprotein integration events, as is common in eutherian mammals. Following this logic, bornavirus replicase (*L* gene) mRNA should be the least abundant, so would be expected to endogenise less frequently as less template is available. However, this study identified that bornavirus replicase-like transcripts were much more prevalent than bornavirus nucleoprotein-like transcripts throughout Australian marsupials (Figs 3 and 4), contrary to what is expected via chance integration.

Other genes including bornavirus matrix, glycoprotein and phosphoprotein, and filovirus VP35 and polymerase, which have been identified throughout eutherian genomes, were not prevalent in marsupial genomes or transcriptomes. This clear bias for filovirus nucleoprotein and bornavirus polymerase genes suggests a selective pressure to endogenise or retain these particular regions of the viral genomes.

### 4.2 EBLL and EFLNP sequences in the koala are accessible templates for small RNA formation

EVEs that accumulate stop codons over time lose their coding functionality within a cell; however, they may retain functionality as non-coding RNA when transcribed. Non-coding RNA arising from EVEs has been implicated in many cellular functions, including inhibition of cancer cell proliferation through cell cycle arrest, maintenance of pluripotency in embryonic stem cells, and antiviral immunity through RNA interference (RNAi) (Xu et al. 2013; Parrish et al. 2015).

Mammals primary immune defence is interferon-based; however, mammalian small RNA retains antiviral potential (Benitez et al. 2015). Consistent with this, antisense piRNA-like molecules arise from EBLN integrations in rodents, suggesting an antiviral protective mechanism (Parrish et al. 2015). In marsupials, piRNAs from retroviral EVEs are transcribed in the koala testis as a possible germline defence (Yu et al. 2019). Additionally, several viruses supress RNAi in mammalian cells, indicating that RNAi suppression improves viral replication (Cui et al. 2015). Thus, the use of small RNA as a host defence should be considered in the context of marsupial antiviral defence.

This study analysed small RNA from koalas and identified both siRNA and piRNA-length molecules derived from non-retroviral EVEs in the koala (Fig. 5). Of the fifteen identified koala non-retroviral EVEs, bornavirus replicase and filovirus nucleoprotein EVEs produced high levels of small RNA (Fig. 5, panel A). These small RNA molecules were enriched in the testis, where the piRNA pathway is most active (Malone and Hannon 2009). We suggest that this pathway is utilised in marsupial germline cells to protect against the integration of *Bornaviridae* and *Filoviridae* sequences.

Unlike in rodents, where piRNA is produced from EBLN integrations, very few of the koala small RNA molecules mapped to the bornavirus nucleoprotein pcEBLN1, indicating that this integration is likely not utilised for small RNA production, and bornavirus replicase integrations are favoured. piRNA molecules can be produced in one of two ways: as primary piRNAs that are loaded into Piwi proteins in most cells or as secondary piRNAs created through ping-pong amplification cycle (Senti and Brennecke 2010). Primary piRNAs are produced as a single strand, which have a 5ʹ uridine bias that helps the Piwi protein bind the RNA (Ozata et al. 2019). In this study, koala EVEs generated primarily unidirectional piRNA-length molecules, which had a 5ʹ uridine bias indicative of primary piRNA and Piwi protein binding (Fig. 5, panel C). A subset of piRNA-length molecules were in the opposite orientation and had both a 5ʹ uridine bias and a 10ʹ adenine bias, characteristic of secondary piRNA created through ping-pong amplification. Additionally, 83.7 per cent of mapping piRNA-length molecules were antisense to EVE transcripts, suggesting that they may be involved in the regulation of transcript levels or protection from further EVE duplication within a genome. Experiments to prove the interactions between small RNA and Piwi-clade proteins would further strengthen this hypothesis.

The prevalence of small RNA derived from select bornavirus replicase and filovirus nucleoprotein EVEs in the koala testis is suggestive of a function, regulatory or protective, for these conserved EVEs. Many other small RNA pathways and molecules exist, including RNA-directed DNA methylation, micro RNAs, and small nucleolar RNAs, which should be considered when investigating the function of these small RNAs, along with the possibility of longer transcripts acting as long non-coding RNAs.

### 4.3 Small RNA in marsupials could protect pouch young from viral infection

In eutherian mammals, small RNAs are involved in post-transcriptional regulation of biological processes, especially during development (Schuster, Miesen, and van Rij 2019). Marsupial development differs greatly from that of eutherian mammals: they are born underdeveloped with a poorly functional immune system (Old and Deane 2000; Goldman 2012). Antiviral defence during development, in the pouch young stage, is reliant on antimicrobial agents secreted in the pouch and mothers’ milk (Sharp et al. 2017).

In the present study, we speculate that marsupials maintained select *Bornaviridae* and *Filoviridae* germline insertions, as a viral defence, to adjunct the developing immune system of marsupial embryos and newborn pouch young. Congruent with this hypothesis of EVE transcripts acting as a viral defence, recent studies have identified that mammalian RNAi protects specialised cells against viral infection (Maillard et al. 2013; Benitez et al. 2015). We suspect other activities, and roles of genomic marsupial EVEs remain undiscovered.

### 4.4 Conclusion

This study provides novel insights into the diversity of non-retroviral EVEs throughout Australian marsupial species. Our analyses describe the trend of frequent *Bornaviridae*, *Filoviridae*, and *Parvoviridae* viral integrations and the overrepresentation of nucleoprotein and replicase viral regions in marsupial genomes and transcriptomes. These trends, whilst similar to eutherian mammals, differ in that bornavirus replicase genes are the most prevalent, rather than bornavirus nucleoprotein integrations that are common throughout Eutherians. The identification of EVE-derived small RNA molecules suggests a protective system utilised throughout marsupial evolution.

## Supplementary Material

veab076_Supp

## Data Availability

All data sets used in this study were publicly available and downloaded from NCBI SRA database. Data sets used are detailed in the Supplementary material.
