# Supplementary material for: Ancient viral integrations in marsupials: a potential antiviral defence
Source: Virus Evol. 2021 Sep 2;7(2):veab076. doi: 10.1093/ve/veab076 (PMC8449507; doi:10.1093/ve/veab076)
Supplement: veab076_Supp [file veab076_supp.zip › Supplementary data Aug25.docx]

**Supplementary data**

Table S1 - *RNA-Seq datasets selected for viral discovery*

Table S2 – *Viral transcripts from marsupial RNA-Sequencing datasets*

Table S3 – *Genomic EVEs identified*

Table S4 – *Small RNA datasets used*

Table S5 – *Small RNA mapped to genomic EVEs and EVE transcripts*

*Supplementary Table S1: RNA-Seq datasets selected for viral discovery*

| **Species** | **Common name** | **Tissue** | **SRA Accession** | **Sequencing instrument** | **Read spots** | **Animal details** |
| --- | --- | --- | --- | --- | --- | --- |
| *Perameles nasuta* | Long-nosed bandicoot | Heart | SRR5840030 | Illumina HiSeq 2500 | 122,723,633 | Wild bandicoot |
| *Perameles nasuta* | Long-nosed bandicoot | Kidney | SRR5840033 | Illumina HiSeq 2500 | 119,786,670 | Wild bandicoot |
| *Perameles nasuta* | Long-nosed bandicoot | Liver | SRR5840031 | Illumina HiSeq 2500 | 124,925,498 | Wild bandicoot |
| *Perameles nasuta* | Long-nosed bandicoot | Spleen | SRR5840032 | Illumina HiSeq 2500 | 114,633,908 | Wild bandicoot |
| *Sminthopsis crassicaudata* | Fat tailed dunnart | Spleen | SRR5097215 | Illumina HiSeq 2500 | 35,546,790 | Adult infected with toxoplasmosis |
| *Sminthopsis crassicaudata* | Fat tailed dunnart | Spleen | SRR5097214 | Illumina HiSeq 2500 | 24,166,064 | Adult |
| *Sminthopsis crassicaudata* | Fat tailed dunnart | Spleen | SRR5097213 | Illumina HiSeq 2500 | 34,510,086 | Adult |
| *Sminthopsis crassicaudata* | Fat tailed dunnart | Uterus | SRR5949851 | Illumina HiSeq 2500 | 34,549,425 | Adult female |
| *Macropus eugenii* | Tammar wallaby | Mammary gland | SRR5074479 | Illumina HiSeq 2000 | 35,745,171 | Adult female |
| *Macropus eugenii* | Tammar wallaby | Mammary gland | SRR5074478 | Illumina HiSeq 2000 | 39,892,227 | Adult female |
| *Macropus eugenii* | Tammar wallaby | Liver | SRR1552212 | Illumina HiSeq 2000 | 47,560,234 | Female from inbred colony |
| *Macropus eugenii* | Tammar wallaby | Skin | SRR831709 | Illumina HiSeq 2000 | 24,828,905 | 1.5 day old infant |
| *Vombatus ursinus* | Bare nosed wombat | Thyroid | ERR2716215 | Illunina HiSeq 4000 | 72,188,191 | Adult |
| *Vombatus ursinus* | Bare nosed wombat | Skin | ERR2716213 | Illunina HiSeq 4000 | 62,550,566 | Adult |
| *Vombatus ursinus* | Bare nosed wombat | Skeletal muscle | ERR2716211 | Illunina HiSeq 4000 | 58,176,962 | Adult |
| *Vombatus ursinus* | Bare nosed wombat | Lymph node | ERR2716209 | Illunina HiSeq 4000 | 62,791,864 | Adult |
| *Sarcophilus harrisii* | Tasmanian devil | Liver | ERR1331711 | Illumina HiSeq 2000 | 34,853,610 | Wild female |
| *Sarcophilus harrisii* | Tasmanian devil | Milk | SRR1793358 | Illumina HiSeq 2000 | 224,654,290 | Zoo-bred female |
| *Sarcophilus harrisii* | Tasmanian devil | PBMC | SRR5420458 | Illumina HiSeq 2000 | 82,254,904 | Adult female with DFTD |
| *Sarcophilus harrisii* | Tasmanian devil | PBMC | SRR5420457 | Illumina HiSeq 2000 | 82,609,965 | Adult female with DFTD |
| *Phascolarctos cinereus* | Koala | Liver | SRR8708137 | Illunina HiSeq 4000 | 105,594,554 | Adult |
| *Phascolarctos cinereus* | Koala | Testis | SRR8708134 | Illunina HiSeq 4000 | 105,701,687 | Adult |
| *Phascolarctos cinereus* | Koala | Brain | SRR8708136 | Illunina HiSeq 4000 | 105,493,875 | Adult |
| *Phascolarctos cinereus* | Koala | PBMC | SRR10375683 | NextSeq 500 | 44,985,875 | Adult male |
| *Petaurus breviceps* | Sugar glider | Brain | SRR636967 | Illunina Genome Analyzer IIx | 19,884,025 | Wild adult |
| *Petaurus breviceps* | Sugar glider | Brain | SRR636966 | Illunina Genome Analyzer IIx | 20,487,681 | Wild adult |
| *Petaurus breviceps* | Sugar glider | Kidney | SRR636921 | Illunina Genome Analyzer IIx | 21,397,068 | Wild adult |
| *Petaurus breviceps* | Sugar glider | Kidney | SRR636922 | Illunina Genome Analyzer IIx | 22,155,124 | Wild adult |
| *Pseudantechinus macdonnellensis* | False antechinus | Liver | SRR3901712 | Illumina HiSeq 2000 | 17,062,977 | - |
| *Isoodon obesulus* | Southern brown bandicoot | Liver | SRR3901713 | Illumina HiSeq 2000 | 19,499,908 | - |
| *Dactylopsila trivirgata* | Striped possum | Liver | SRR3901714 | Illumina HiSeq 2000 | 16,856,502 | - |
| *Trichosurus vulpecula* | Brushtail possum | Liver | SRR3901715 | Illumina HiSeq 2000 | 15,601,079 | - |
| *Cercartetus concinnus* | Western Pygmy possum | Liver | SRR3901718 | Illumina HiSeq 2000 | 14,355,693 | - |
| *Petrogale xanthopus* | Yellow footed rock wallaby | Liver | SRR3901717 | Illumina HiSeq 2000 | 19,694,753 | - |

*Supplementary Table S2: Viral transcripts identified from Australian marsupial RNA-Sequencing datasets*

| ***Dataset details*** | | |  |  | ***Closest viral hit*** | | | | | | |
| --- | --- | --- | --- | --- | --- | --- | --- | --- | --- | --- | --- |
| ***Scientific name*** | ***Common name*** | ***Dataset*** | ***EVE Criteria**** | ***Reads*** | ***Closest viral relative (BLASTx)*** | ***% pairwise identity (AA)*** | ***E-value*** | ***Accession*** | ***HSP len AA*** | ***Family*** | ***Host*** |
| *Pseudantechinus macdonnellensis* | *False antechinus* | *SRR3901712* | *E* | *12* | *N [Southwest carpet python virus]* | *35.70%* | *1.15E-21* | *YP_009508485* | *111* | *Bornaviridae* | *reptile* |
| *Isoodon obesulus* | *Southern brown bandicoot* | *SRR3901713* | *N/A* |  | *polymerase [parrot hepatitis B virus]* | *47.50%* | *1.33E-05* | *AFY97722* | *57* | *Hepadnaviridae* | *bird* |
| *Isoodon obesulus* | *Southern brown bandicoot* | *SRR3901713* | *N/A* |  | *polymerase [Sheldgoose hepatitis B virus]* | *54.60%* | *3.41E-34* | *YP_024974* | *107* | *Hepadnaviridae* | *bird* |
| *Perameles nasuta* | *Long nosed bandicoot* | *SRR5840030* | *id, E* | *21* | *N [Southwest carpet python virus]* | *47.40%* | *1.18E-08* | *YP_009508485* | *77* | *Bornaviridae* | *reptile* |
| *Perameles nasuta* | *Long nosed bandicoot* | *SRR5840030* | *E* | *218* | *phosphoprotein [Canary bornavirus 2]* | *80.00%* | *4.02E-61* | *AGJ74916* | *64* | *Bornaviridae* | *bird* |
| *Perameles nasuta* | *Long nosed bandicoot* | *SRR5840030* | *E* | *73* | *L [Southwest carpet python virus]* | *37.10%* | *4.09E-09* | *YP_009508490* | *110* | *Bornaviridae* | *reptile* |
| *Perameles nasuta* | *Long nosed bandicoot* | *SRR5840030* | *sh, E* | *75* | *RNA-dependent RNA polymerase [Borna disease virus 2]* | *52.50%* | *2.48E-10* | *YP_009268922* | *58* | *Bornaviridae* | *mammal* |
| *Perameles nasuta* | *Long nosed bandicoot* | *SRR5840033* | *E* | *164* | *RNA-dependent RNA polymerase [Borna disease virus 2]* | *48.60%* | *1.76E-10* | *YP_009268922* | *71* | *Bornaviridae* | *mammal* |
| *Perameles nasuta* | *Long nosed bandicoot* | *SRR5840033* | *sc, E* | *14* | *endogenous bornavirus-like L-1 [Eptesicus serotinus]* | *46.50%* | *2.00E-17* | *BAV60925* | *98* | *Bornaviridae* | *mammal* |
| *Perameles nasuta* | *Long nosed bandicoot* | *SRR5840033* | *sc, E* | *34* | *N [Southwest carpet python virus]* | *47.40%* | *8.72E-10* | *YP_009508485* | *77* | *Bornaviridae* | *reptile* |
| *Perameles nasuta* | *Long nosed bandicoot* | *SRR5840033* | *sc, E* | *5* | *capsid protein VP1 [Canine parvovirus]* | *50.00%* | *1.97E-20* | *QBO24456* | *93* | *Parvoviridae* | *mammal* |
| *Perameles nasuta* | *Long nosed bandicoot* | *SRR5840033* | *E* | *6* | *putative VP1 [Tusavirus 1]* | *47.40%* | *2.26E-09* | *AIT18930* | *56* | *Parvoviridae* | *mammal* |
| *Perameles nasuta* | *Long nosed bandicoot* | *SRR5840033* | *E* | *11* | *endogenous bornavirus-like L-1 [Eptesicus nilssonii]* | *52.40%* | *0.000112* | *BAV60921* | *20* | *Bornaviridae* | *mammal* |
| *Perameles nasuta* | *Long nosed bandicoot* | *SRR5840033* | *E* | *11461* | *L [Jungle carpet python virus]* | *29.80%* | *2.17E-31* | *YP_009508484* | *449* | *Bornaviridae* | *reptile* |
| *Perameles nasuta* | *Long nosed bandicoot* | *SRR5840033* | *E* | *268* | *phosphoprotein [Canary bornavirus 2]* | *80.00%* | *4.02E-61* | *AGJ74916* | *64* | *Bornaviridae* | *bird* |
| *Perameles nasuta* | *Long nosed bandicoot* | *SRR5840033* | *sh, sc, E* | *4* | *L protein [Wuhan sharpbelly bornavirus]* | *71.90%* | *3.60E-08* | *AVM87541* | *31* | *Bornaviridae* | *fish* |
| *Perameles nasuta* | *Long nosed bandicoot* | *SRR5840031* | *E* | *229* | *RNA-dependent RNA polymerase [Borna disease virus 2]* | *48.60%* | *1.76E-10* | *YP_009268922* | *71* | *Bornaviridae* | *mammal* |
| *Perameles nasuta* | *Long nosed bandicoot* | *SRR5840031* | *E* | *1722* | *phosphoprotein [Canary bornavirus 2]* | *78.50%* | *1.19E-60* | *AGJ74916* | *64* | *Bornaviridae* | *bird* |
| *Perameles nasuta* | *Long nosed bandicoot* | *SRR5840031* | *sc, E* | *52* | *L [Southwest carpet python virus]* | *36.20%* | *6.51E-08* | *YP_009508490* | *110* | *Bornaviridae* | *reptile* |
| *Perameles nasuta* | *Long nosed bandicoot* | *SRR5840032* | *sc, id, E* | *19* | *N [Southwest carpet python virus]* | *46.20%* | *3.25E-08* | *YP_009508485* | *77* | *Bornaviridae* | *reptile* |
| *Perameles nasuta* | *Long nosed bandicoot* | *SRR5840032* | *E* | *2515* | *RNA-dependent RNA polymerase [Borna disease virus 2]* | *48.60%* | *1.76E-10* | *YP_009268922* | *71* | *Bornaviridae* | *mammal* |
| *Perameles nasuta* | *Long nosed bandicoot* | *SRR5840032* | *sc, E* | *468* | *L [Jungle carpet python virus]* | *29.80%* | *2.17E-31* | *YP_009508484* | *449* | *Bornaviridae* | *reptile* |
| *Perameles nasuta* | *Long nosed bandicoot* | *SRR5840032* | *E* | *216* | *phosphoprotein [Canary bornavirus 2]* | *80.00%* | *2.00E-61* | *AGJ74916* | *64* | *Bornaviridae* | *bird* |
| *Perameles nasuta* | *Long nosed bandicoot* | *SRR5840032* | *sc, E* | *1989* | *nucleocapsid protein [Mengla dianlovirus]* | *40.90%* | *4.27E-14* | *AQM57739* | *92* | *Filoviridae* | *mammal* |
| *Perameles nasuta* | *Long nosed bandicoot* | *SRR5840032* | *sc, E* | *41* | *endogenous Bornavirus-like nucleoprotein 1 [Miniopterus natalensis]* | *37.80%* | *2.02E-16* | *XP_016072469* | *147* | *Bornaviridae* | *mammal* |
| *Sminthopsis crassicaudata* | *Fat tailed dunnart* | *SRR5097215* | *id, E* | *131* | *N [Southwest carpet python virus]* | *33.50%* | *1.44E-25* | *YP_009508485* | *166* | *Bornaviridae* | *reptile* |
| *Sminthopsis crassicaudata* | *Fat tailed dunnart* | *SRR5097214* | *id, E* | *38* | *Polymerase [Borna disease virus 1]* | *48.40%* | *3.77E-09* | *CAC70645* | *63* | *Bornaviridae* | *mammal* |
| *Sminthopsis crassicaudata* | *Fat tailed dunnart* | *SRR5097214* | *sc, E* | *281* | *N [Southwest carpet python virus]* | *28.40%* | *2.93E-32* | *YP_009508485* | *321* | *Bornaviridae* | *reptile* |
| *Sminthopsis crassicaudata* | *Fat tailed dunnart* | *SRR5097214* | *sc, E* | *24* | *RNA-dependent RNA polymerase [Parrot bornavirus 7]* | *41.50%* | *3.29E-11* | *YP_009268904* | *116* | *Bornaviridae* | *bird* |
| *Sminthopsis crassicaudata* | *Fat tailed dunnart* | *SRR5097213* | *sc, E* | *45* | *RNA-dependent RNA polymerase [Parrot bornavirus 7]* | *58.60%* | *2.84E-15* | *YP_009268904* | *28* | *Bornaviridae* | *bird* |
| *Sminthopsis crassicaudata* | *Fat tailed dunnart* | *SRR5097213* | *id, sc, E* | *264* | *N [Southwest carpet python virus]* | *29.00%* | *1.00E-29* | *YP_009508485* | *273* | *Bornaviridae* | *reptile* |
| *Sminthopsis crassicaudata* | *Fat tailed dunnart* | *SRR5097213* | *sc, E* | *534* | *capsid protein [Bearded dragon parvovirus]* | *53.30%* | *3.45E-11* | *YP_009154713* | *59* | *Parvoviridae* | *reptile* |
| *Sminthopsis crassicaudata* | *Fat tailed dunnart* | *SRR5097213* | *id, E* | *87* | *L-polymerase [Variegated squirrel bornavirus 1]* | *42.20%* | *1.44E-09* | *SBT82903* | *89* | *Bornaviridae* | *mammal* |
| *Sminthopsis crassicaudata* | *Fat tailed dunnart* | *SRR5949851* | *id, E* | *405* | *N [Southwest carpet python virus]* | *28.40%* | *2.95E-32* | *YP_009508485* | *321* | *Bornaviridae* | *reptile* |
| *Sminthopsis crassicaudata* | *Fat tailed dunnart* | *SRR5949851* | *E* | *211* | *L-polymerase [Variegated squirrel bornavirus 1]* | *43.30%* | *3.59E-10* | *SBT82897* | *89* | *Bornaviridae* | *mammal* |
| *Sminthopsis crassicaudata* | *Fat tailed dunnart* | *SRR5949851* | *E* | *66* | *RNA-dependent RNA polymerase [Parrot bornavirus 7]* | *58.60%* | *6.27E-15* | *YP_009268904* | *28* | *Bornaviridae* | *bird* |
| *Petaurus breviceps* | *Sugar glider* | *SRR636967* | *id, E* | *69* | *nucleoprotein [Reston ebolavirus]* | *39.00%* | *2.10E-10* | *AUD54831* | *76* | *Filoviridae* | *mammal* |
| *Petaurus breviceps* | *Sugar glider* | *SRR636967* | *id, E* | *102* | *NP [Zaire ebolavirus]* | *52.80%* | *7.25E-42* | *AJB84693* | *143* | *Filoviridae* | *mammal* |
| *Petaurus breviceps* | *Sugar glider* | *SRR636967* | *E* | *174* | *matrix protein, partial [Parrot bornavirus 4]* | *41.50%* | *4.93E-08* | *AZW33651* | *81* | *Bornaviridae* | *bird* |
| *Petaurus breviceps* | *Sugar glider* | *SRR636967* | *id, sh, E* | *13* | *RNA-dependent RNA polymerase [Aquatic bird bornavirus 2]* | *44.90%* | *0.000424* | *YP_009268916* | *48* | *Bornaviridae* | *bird* |
| *Petaurus breviceps* | *Sugar glider* | *SRR636967* | *sh, sc, E* | *28* | *L polymerase [Loveridges garter snake virus 1]* | *58.50%* | *1.02E-16* | *YP_009055063* | *64* | *Bornaviridae* | *reptile* |
| *Petaurus breviceps* | *Sugar glider* | *SRR636967* | *id, E* | *91* | *major nucleoprotein, partial [Marburg marburgvirus]* | *48.40%* | *4.54E-17* | *AIX02912* | *93* | *Filoviridae* | *mammal* |
| *Petaurus breviceps* | *Sugar glider* | *SRR636966* | *E* | *147* | *major nucleoprotein, partial [Marburg marburgvirus]* | *48.40%* | *3.42E-17* | *AIX02912* | *93* | *Filoviridae* | *mammal* |
| *Petaurus breviceps* | *Sugar glider* | *SRR636966* | *E* | *197* | *matrix protein, partial [Parrot bornavirus 4]* | *41.50%* | *4.93E-08* | *AZW33651* | *81* | *Bornaviridae* | *bird* |
| *Petaurus breviceps* | *Sugar glider* | *SRR636966* | *id, sh, E* | *66* | *nucleoprotein, partial [Lloviu cuevavirus]* | *61.80%* | *9.25E-21* | *AWC68490* | *75* | *Filoviridae* | *mammal* |
| *Petaurus breviceps* | *Sugar glider* | *SRR636966* | *id, E* | *28* | *nucleoprotein [Marburg virus - Musoke, Kenya, 1980]* | *46.20%* | *5.51E-06* | *CAA78114* | *64* | *Filoviridae* | *mammal* |
| *Petaurus breviceps* | *Sugar glider* | *SRR636921* | *id, E* | *51* | *N [Sudan virus - Boniface, Sudan,1976]* | *50.60%* | *5.70E-11* | *Q9QP77* | *80* | *Filoviridae* | *mammal* |
| *Petaurus breviceps* | *Sugar glider* | *SRR636921* | *id, E* | *134* | *nucleoprotein [Reston ebolavirus]* | *36.40%* | *5.35E-15* | *AAV48574* | *128* | *Filoviridae* | *mammal* |
| *Petaurus breviceps* | *Sugar glider* | *SRR636921* | *id, E* | *96* | *major nucleoprotein, partial [Marburg marburgvirus]* | *57.80%* | *1.55E-16* | *AIX02912* | *62* | *Filoviridae* | *mammal* |
| *Petaurus breviceps* | *Sugar glider* | *SRR636921* | *E* | *185* | *matrix protein, partial [Parrot bornavirus 4]* | *41.50%* | *1.43E-08* | *AZW33651* | *81* | *Bornaviridae* | *bird* |
| *Petaurus breviceps* | *Sugar glider* | *SRR636921* | *E* | *600* | *glycoprotein [Wuhan sharpbelly bornavirus]* | *32.40%* | *2.02E-19* | *AVM87539* | *209* | *Bornaviridae* | *fish* |
| *Petaurus breviceps* | *Sugar glider* | *SRR636922* | *E* | *191* | *matrix protein, partial [Parrot bornavirus 4]* | *41.50%* | *1.43E-08* | *AZW33651* | *81* | *Bornaviridae* | *bird* |
| *Petaurus breviceps* | *Sugar glider* | *SRR636922* | *E* | *430* | *glycoprotein [Wuhan sharpbelly bornavirus]* | *32.40%* | *2.02E-19* | *AVM87539* | *209* | *Bornaviridae* | *fish* |
| *Petaurus breviceps* | *Sugar glider* | *SRR636922* | *id, E* | *51* | *nucleoprotein [Bundibugyo ebolavirus]* | *48.50%* | *4.34E-15* | *YP_003815432* | *102* | *Filoviridae* | *mammal* |
| *Petaurus breviceps* | *Sugar glider* | *SRR636922* | *sc, E* | *232* | *nucleoprotein [Reston ebolavirus]* | *36.40%* | *3.10E-14* | *AAV48574* | *128* | *Filoviridae* | *mammal* |
| *Petaurus breviceps* | *Sugar glider* | *SRR636922* | *id, E* | *32* | *major nucleoprotein, partial [Marburg marburgvirus]* | *53.80%* | *8.68E-08* | *AIX02912* | *50* | *Filoviridae* | *mammal* |
| *Petaurus breviceps* | *Sugar glider* | *SRR636922* | *id, E* | *163* | *NP, partial [Zaire ebolavirus]* | *52.00%* | *5.07E-50* | *AJB84693* | *174* | *Filoviridae* | *mammal* |
| *Phascolarctos cinereus* | *koala* | *SRR8708136* | *G* |  | *Polymerase [Borna disease virus 1]* | *54.80%* | *1.81E-15* | *CAC70645* | *72* | *Bornaviridae* | *mammal* |
| *Phascolarctos cinereus* | *koala* | *SRR8708136* | *G* |  | *RNA-dependent RNA polymerase [Parrot bornavirus 7]* | *27.10%* | *1.05E-05* | *YP_009268904* | *216* | *Bornaviridae* | *bird* |
| *Phascolarctos cinereus* | *koala* | *SRR8708136* | *G* |  | *L polymerase [Loveridges garter snake virus 1]* | *56.90%* | *6.04E-100* | *YP_009055063* | *71* | *Bornaviridae* | *reptile* |
| *Phascolarctos cinereus* | *koala* | *SRR8708136* | *G* |  | *nucleoprotein [Marburg virus - Musoke, Kenya, 1980]* | *40.90%* | *6.61E-40* | *CAA78114* | *231* | *Filoviridae* | *mammal* |
| *Phascolarctos cinereus* | *koala* | *SRR8708136* | *G* |  | *nucleocapsid protein [Mengla dianlovirus]* | *38.20%* | *5.98E-37* | *AQM57739* | *227* | *Filoviridae* | *mammal* |
| *Phascolarctos cinereus* | *koala* | *SRR8708136* | *G* |  | *non-structural protein 1 [Goose parvovirus]* | *64.60%* | *2.22E-12* | *APB54219* | *47* | *Parvoviridae* | *bird* |
| *Phascolarctos cinereus* | *koala* | *SRR8708137* | *G* |  | *putative nucleoprotein [Guangdong red-banded snake chuvirus-like virus]* | *42.90%* | *1.28E-08* | *AVM87274* | *62* | *RNA* | *reptile* |
| *Phascolarctos cinereus* | *koala* | *SRR8708137* | *G* |  | *VP2 protein [Porcine parvovirus]* | *44.90%* | *1.72E-09* | *AHB30931* | *84* | *Parvoviridae* | *mammal* |
| *Phascolarctos cinereus* | *koala* | *SRR8708137* | *G* |  | *nucleocapsid protein [Mengla dianlovirus]* | *38.20%* | *2.59E-36* | *AQM57739* | *227* | *Filoviridae* | *mammal* |
| *Phascolarctos cinereus* | *koala* | *SRR8708137* | *G* |  | *L polymerase [Loveridges garter snake virus 1]* | *58.30%* | *1.03E-85* | *YP_009055063* | *71* | *Bornaviridae* | *reptile* |
| *Phascolarctos cinereus* | *koala* | *SRR8708137* | *G* |  | *VP1 capsid protein, partial [Cutavirus]* | *50.00%* | *0.000328* | *QGL40397* | *41* | *Parvoviridae* | *mammal* |
| *Phascolarctos cinereus* | *koala* | *SRR8708137* | *G* |  | *RNA-dependent RNA polymerase [Parrot bornavirus 7]* | *27.10%* | *2.60E-06* | *YP_009268904* | *216* | *Bornaviridae* | *bird* |
| *Phascolarctos cinereus* | *koala* | *SRR8708137* | *G* |  | *nucleoprotein [Marburg virus - Musoke, Kenya, 1980]* | *40.90%* | *6.68E-40* | *CAA78114* | *231* | *Filoviridae* | *mammal* |
| *Phascolarctos cinereus* | *koala* | *SRR8708137* | *G* |  | *putative nucleoprotein [Guangdong red-banded snake chuvirus-like virus]* | *36.40%* | *1.50E-07* | *AVM87274* | *76* | *RNA* | *reptile* |
| *Phascolarctos cinereus* | *koala* | *SRR10375683* | *G* |  | *nucleoprotein [Reston ebolavirus - Reston]* | *62.20%* | *7.58E-05* | *ACT22784* | *36* | *Filoviridae* | *mammal* |
| *Phascolarctos cinereus* | *koala* | *SRR10375683* | *G* |  | *nucleoprotein, partial [Reston ebolavirus]* | *49.20%* | *1.30E-12* | *AUD54831* | *64* | *Filoviridae* | *mammal* |
| *Phascolarctos cinereus* | *koala* | *SRR10375683* | *G* |  | *nucleoprotein [Reston ebolavirus]* | *60.00%* | *0.00049* | *AAV48574* | *34* | *Filoviridae* | *mammal* |
| *Phascolarctos cinereus* | *koala* | *SRR10375683* | *G* |  | *major nucleoprotein, partial [Marburg marburgvirus]* | *57.10%* | *6.07E-11* | *AIX02912* | *48* | *Filoviridae* | *mammal* |
| *Phascolarctos cinereus* | *koala* | *SRR10375683* | *G* |  | *RNA-dependent RNA polymerase [Parrot bornavirus 7]* | *28.70%* | *9.55E-06* | *YP_009268904* | *165* | *Bornaviridae* | *bird* |
| *Phascolarctos cinereus* | *koala* | *SRR10375683* | *G* |  | *L protein [Aquatic bird bornavirus 1]* | *60.60%* | *5.68E-33* | *QGZ07388* | *70* | *Bornaviridae* | *bird* |
| *Phascolarctos cinereus* | *koala* | *SRR10375683* | *G* |  | *VP2 protein [Porcine parvovirus]* | *44.90%* | *4.73E-10* | *AHB30931* | *84* | *Parvoviridae* | *mammal* |
| *Phascolarctos cinereus* | *koala* | *SRR10375683* | *G* |  | *VP2, partial [Bat parvovirus]* | *25.90%* | *8.66E-11* | *AIF74208* | *259* | *Parvoviridae* | *mammal* |
| *Phascolarctos cinereus* | *koala* | *SRR10375683* | *G* |  | *VP2, partial [Myotis ricketti parvovirus]* | *27.20%* | *2.85E-11* | *AGU69468* | *111* | *Parvoviridae* | *mammal* |
| *Phascolarctos cinereus* | *koala* | *SRR10375683* | *G* |  | *VP1 capsid protein, partial [Cutavirus]* | *52.40%* | *1.84E-05* | *QGL40397* | *41* | *Parvoviridae* | *mammal* |
| *Phascolarctos cinereus* | *koala* | *SRR10375683* | *G* |  | *capsid protein, partial [Parvovirus fur seal/ATROP40/BR/2012]* | *51.20%* | *0.000764* | *AKI82154* | *40* | *Parvoviridae* | *mammal* |
| *Phascolarctos cinereus* | *koala* | *SRR10375683* | *G* |  | *non-structural protein 1 [Goose parvovirus]* | *64.60%* | *5.13E-13* | *APB54219* | *47* | *Parvoviridae* | *bird* |
| *Phascolarctos cinereus* | *koala* | *SRR10375683* | *G* |  | *L protein [Wuhan sharpbelly bornavirus]* | *46.30%* | *1.47E-05* | *AVM87541* | *40* | *Bornaviridae* | *fish* |
| *Phascolarctos cinereus* | *koala* | *SRR8708134* | *G* |  | *nucleocapsid protein [Mengla dianlovirus]* | *38.20%* | *6.05E-37* | *AQM57739* | *227* | *Filoviridae* | *mammal* |
| *Phascolarctos cinereus* | *koala* | *SRR8708134* | *G* |  | *putative capsid protein, partial [Parvovirus fur seal/AAUST54/BR/2012]* | *43.80%* | *2.51E-18* | *AKI82150* | *104* | *Parvoviridae* | *mammal* |
| *Phascolarctos cinereus* | *koala* | *SRR8708134* | *G* |  | *putative VP1 [Bufavirus-3]* | *78.60%* | *7.95E-10* | *BAO56911* | *13* | *Parvoviridae* | *mammal* |
| *Phascolarctos cinereus* | *koala* | *SRR8708134* | *G* |  | *VP2 protein, partial [Aleutian mink disease virus]* | *38.50%* | *9.74E-32* | *APB42238* | *227* | *Parvoviridae* | *mammal* |
| *Phascolarctos cinereus* | *koala* | *SRR8708134* | *G* |  | *L [Jungle carpet python virus] [Jungle carpet python virus]* | *50.00%* | *8.08E-14* | *YP_009508484* | *77* | *Bornaviridae* | *reptile* |
| *Phascolarctos cinereus* | *koala* | *SRR8708134* | *G* |  | *major nucleoprotein, partial [Marburg marburgvirus]* | *55.80%* | *4.72E-07* | *AIX02912* | *42* | *Filoviridae* | *mammal* |
| *Phascolarctos cinereus* | *koala* | *SRR8708134* | *G* |  | *nucleocapsid protein [Mengla dianlovirus]* | *38.00%* | *5.15E-23* | *AQM57739* | *170* | *Filoviridae* | *mammal* |
| *Phascolarctos cinereus* | *koala* | *SRR8708134* | *G* |  | *VP1 [Miniopterus schreibersii parvovirus]* | *40.00%* | *7.98E-18* | *AGU69470* | *98* | *Parvoviridae* | *mammal* |
| *Phascolarctos cinereus* | *koala* | *SRR8708134* | *G* |  | *viral protein 1, partial [Bat parvovirus BtBV_V3]* | *44.20%* | *3.06E-07* | *AKM21309* | *74* | *Parvoviridae* | *mammal* |
| *Phascolarctos cinereus* | *koala* | *SRR8708134* | *G* |  | *Vp35 [Reston ebolavirus - Reston]* | *41.30%* | *5.96E-15* | *3KS4_A* | *79* | *Filoviridae* | *mammal* |
| *Phascolarctos cinereus* | *koala* | *SRR8708134* | *G* |  | *Polymerase [Borna disease virus 1]* | *54.80%* | *1.83E-15* | *CAC70645* | *72* | *Bornaviridae* | *mammal* |
| *Phascolarctos cinereus* | *koala* | *SRR8708134* | *G* |  | *Polymerase [Borna disease virus 1]* | *54.80%* | *1.83E-15* | *CAC70645* | *72* | *Bornaviridae* | *mammal* |
| *Phascolarctos cinereus* | *koala* | *SRR8708134* | *G* |  | *VP2 protein [Porcine parvovirus]* | *44.90%* | *5.48E-08* | *AHB30931* | *84* | *Parvoviridae* | *mammal* |
| *Phascolarctos cinereus* | *koala* | *SRR8708134* | *G* |  | *NS1, partial [Goat protoparvovirus]* | *57.70%* | *6.87E-14* | *AYN07241* | *25* | *Parvoviridae* | *mammal* |
| *Phascolarctos cinereus* | *koala* | *SRR8708134* | *G* |  | *VP1 [Fox parvovirus]* | *43.90%* | *0.000192* | *AGK45550* | *55* | *Parvoviridae* | *mammal* |
| *Phascolarctos cinereus* | *koala* | *SRR8708134* | *G* |  | *NS1, partial [Goat protoparvovirus]* | *57.70%* | *6.87E-14* | *AYN07241* | *25* | *Parvoviridae* | *mammal* |
| *Phascolarctos cinereus* | *koala* | *SRR8708134* | *G* |  | *rep protein [Bat adeno-associated virus YNM]* | *64.60%* | *1.12E-13* | *YP_003858571* | *47* | *Parvoviridae* | *mammal* |
| *Phascolarctos cinereus* | *koala* | *SRR8708134* | *G* |  | *nucleoprotein, partial [Parrot bornavirus 4]* | *29.60%* | *0.000418* | *QGT41038* | *97* | *Bornaviridae* | *bird* |
| *Phascolarctos cinereus* | *koala* | *SRR8708134* | *G* |  | *L polymerase [Loveridges garter snake virus 1]* | *60.00%* | *2.65E-100* | *YP_009055063* | *64* | *Bornaviridae* | *reptile* |
| *Phascolarctos cinereus* | *koala* | *SRR8708134* | *G* |  | *polymerase [Parrot bornavirus 5]* | *53.20%* | *2.62E-08* | *YP_009512939* | *46* | *Bornaviridae* | *bird* |
| *Phascolarctos cinereus* | *koala* | *SRR8708134* | *G* |  | *nucleoprotein [Marburg virus - Musoke, Kenya, 1980]* | *40.90%* | *6.68E-40* | *CAA78114* | *231* | *Filoviridae* | *mammal* |
| *Phascolarctos cinereus* | *koala* | *SRR8708134* | *G* |  | *major nucleoprotein, partial [Marburg marburgvirus]* | *55.80%* | *5.57E-12* | *AIX02911* | *51* | *Filoviridae* | *mammal* |
| *Phascolarctos cinereus* | *koala* | *SRR8708134* | *G* |  | *VP1 [Canine parvovirus]* | *47.80%* | *9.57E-35* | *QFF91975* | *66* | *Parvoviridae* | *mammal* |
| *Sarcophilus harrisii* | *Tasmanian devil* | *ERR1331711* | *G* |  | *VP1, partial [Bat parvovirus]* | *50.00%* | *1.16E-05* | *AIF74206* | *43* | *Parvoviridae* | *mammal* |
| *Sarcophilus harrisii* | *Tasmanian devil* | *ERR1331711* | *G* |  | *RNA-dependent RNA polymerase [Parrot bornavirus 7]* | *38.50%* | *1.82E-21* | *YP_009268904* | *95* | *Bornaviridae* | *bird* |
| *Sarcophilus harrisii* | *Tasmanian devil* | *ERR1331711* | *G* |  | *N [Jungle carpet python virus]* | *35.10%* | *2.20E-12* | *YP_009508479* | *113* | *Bornaviridae* | *reptile* |
| *Sarcophilus harrisii* | *Tasmanian devil* | *ERR1331711* | *G* |  | *L [Jungle carpet python virus]* | *59.30%* | *2.05E-07* | *YP_009508484* | *26* | *Bornaviridae* | *reptile* |
| *Sarcophilus harrisii* | *Tasmanian devil* | *ERR1331711* | *G* |  | *L polymerase [Loveridges garter snake virus 1]* | *53.60%* | *2.38E-07* | *YP_009055063* | *55* | *Bornaviridae* | *reptile* |
| *Sarcophilus harrisii* | *Tasmanian devil* | *ERR1331711* | *G* |  | *N [Southwest carpet python virus]* | *50.00%* | *9.00E-18* | *YP_009508485* | *77* | *Bornaviridae* | *reptile* |
| *Sarcophilus harrisii* | *Tasmanian devil* | *SRR1793358* | *G* |  | *capsid protein VP2, partial [Aleutian mink disease virus]* | *62.50%* | *1.02E-14* | *ACC95819* | *47* | *Parvoviridae* | *mammal* |
| *Sarcophilus harrisii* | *Tasmanian devil* | *SRR1793358* | *G* |  | *N [Southwest carpet python virus]* | *50.90%* | *1.91E-12* | *YP_009508485* | *56* | *Bornaviridae* | *reptile* |
| *Sarcophilus harrisii* | *Tasmanian devil* | *SRR1793358* | *G* |  | *RNA-dependent RNA polymerase [Canary bornavirus 2]* | *36.30%* | *1.43E-24* | *YP_009165497* | *90* | *Bornaviridae* | *bird* |
| *Sarcophilus harrisii* | *Tasmanian devil* | *SRR1793358* | *G* |  | *NS1, partial [Cutavirus]* | *59.40%* | *0.000155* | *AMS35085* | *31* | *Parvoviridae* | *mammal* |
| *Sarcophilus harrisii* | *Tasmanian devil* | *SRR1793358* | *G* |  | *N [Southwest carpet python virus]* | *34.10%* | *1.40E-19* | *YP_009508485* | *166* | *Bornaviridae* | *reptile* |
| *Sarcophilus harrisii* | *Tasmanian devil* | *SRR5420458* | *G* |  | *ORF58 [Bovine gammaherpesvirus 6]* | *31.50%* | *8.24E-48* | *YP_009042037* | *297* | *Herpesviridae* | *mammal* |
| *Sarcophilus harrisii* | *Tasmanian devil* | *SRR5420458* | *G* |  | *transcriptional control protein [Bovine gammaherpesvirus 6]* | *35.10%* | *1.95E-13* | *YP_009042036* | *132* | *Herpesviridae* | *mammal* |
| *Sarcophilus harrisii* | *Tasmanian devil* | *SRR5420458* | *G* |  | *L, partial [Parrot bornavirus 4]* | *40.20%* | *5.39E-06* | *ACJ71393* | *81* | *Bornaviridae* | *bird* |
| *Sarcophilus harrisii* | *Tasmanian devil* | *SRR5420458* | *G* |  | *N [Southwest carpet python virus]* | *34.10%* | *3.04E-39* | *YP_009508485* | *166* | *Bornaviridae* | *reptile* |
| *Sarcophilus harrisii* | *Tasmanian devil* | *SRR5420458* | *G* |  | *L [Southwest carpet python virus]* | *45.30%* | *6.26E-07* | *YP_009508490* | *62* | *Bornaviridae* | *reptile* |
| *Sarcophilus harrisii* | *Tasmanian devil* | *SRR5420458* | *G* |  | *RNA-dependent RNA polymerase [Parrot bornavirus 7]* | *37.60%* | *1.79E-07* | *YP_009268904* | *133* | *Bornaviridae* | *bird* |
| *Sarcophilus harrisii* | *Tasmanian devil* | *SRR5420457* | *N/A* |  | *UL49 [Phascolarctid gammaherpesvirus 1]* | *42.40%* | *1.51E-10* | *AZB49238* | *65* | *Herpesviridae* | *mammal* |
| *Sarcophilus harrisii* | *Tasmanian devil* | *SRR5420457* | *N/A* |  | *ORF58 [Bovine gammaherpesvirus 6]* | *32.40%* | *9.59E-49* | *YP_009042037* | *338* | *Herpesviridae* | *mammal* |
| *Sarcophilus harrisii* | *Tasmanian devil* | *SRR5420457* | *N/A* |  | *multifunctional expression regulator [Rhinolophus gammaherpesvirus 1]* | *33.70%* | *6.73E-09* | *YP_009551870* | *82* | *Herpesviridae* | *mammal* |
| *Sarcophilus harrisii* | *Tasmanian devil* | *SRR5420457* | *N/A* |  | *transcriptional control protein [Bovine gammaherpesvirus 6]* | *35.90%* | *6.73E-11* | *YP_009042036* | *115* | *Herpesviridae* | *mammal* |
| *Sarcophilus harrisii* | *Tasmanian devil* | *SRR5420457* | *N/A* |  | *ORF2 [Torque teno Leptonychotes weddellii virus-1]* | *64.50%* | *1.39E-07* | *ASA48606* | *30* | *Anelloviridae* | *mammal* |
| *Sarcophilus harrisii* | *Tasmanian devil* | *SRR5420457* | *G* |  | *N [Southwest carpet python virus]* | *34.10%* | *3.04E-39* | *YP_009508485* | *166* | *Bornaviridae* | *reptile* |
| *Sarcophilus harrisii* | *Tasmanian devil* | *SRR5420457* | *N/A* |  | *ORF1, partial [Torque teno virus]* | *34.40%* | *7.38E-09* | *QBA83988* | *91* | *Anelloviridae* | *mammal* |
| *Sarcophilus harrisii* | *Tasmanian devil* | *SRR5420457* | *G* |  | *RNA-dependent RNA polymerase [Canary bornavirus 2]* | *36.30%* | *1.41E-24* | *YP_009165497* | *90* | *Bornaviridae* | *bird* |
| *Sarcophilus harrisii* | *Tasmanian devil* | *SRR5420457* | *G* |  | *L polymerase [Loveridges garter snake virus 1]* | *40.70%* | *5.31E-11* | *YP_009055063* | *107* | *Bornaviridae* | *reptile* |
| *Dactylopsila trivirgata* | *Striped possum* | *SRR3901714* | *E* | *26* | *nucleoprotein [Tai Forest ebolavirus]* | *46.70%* | *5.71E-33* | *YP_003815423* | *89* | *Filoviridae* | *mammal* |
| *Dactylopsila trivirgata* | *Striped possum* | *SRR3901714* | *sc, E* | *12* | *Nucleoprotein [Lake Victoria marburgvirus - Ozolin]* | *41.70%* | *1.90E-18* | *5XSQ_A* | *142* | *Filoviridae* | *mammal* |
| *Cercartetus concinnus* | *Western pygmy possum* | *SRR3901718* | *sc, E* | *39* | *nucleoprotein [Marburg virus - Musoke, Kenya, 1980]* | *33.10%* | *2.65E-21* | *CAA78114* | *178* | *Filoviridae* | *mammal* |
| *Cercartetus concinnus* | *Western pygmy possum* | *SRR3901718* | *E* | *76* | *major nucleoprotein, partial [Marburg marburgvirus]* | *54.70%* | *1.27E-10* | *AIX02912* | *62* | *Filoviridae* | *mammal* |
| *Cercartetus concinnus* | *Western pygmy possum* | *SRR3901718* | *E* | *52* | *nucleoprotein [Zaire ebolavirus]* | *31.40%* | *2.76E-21* | *ALG02095* | *171* | *Filoviridae* | *mammal* |
| *Trichosurus vulpecula* | *Brushtail possum* | *SRR3901715* | *G* |  | *nucleoprotein [Reston ebolavirus - Reston]* | *40.00%* | *9.91E-36* | *ACT22784* | *143* | *Filoviridae* | *mammal* |
| *Notamacropus eugenii* | *Tammar wallaby* | *SRR5074479* | *G* |  | *NS1, partial [Mfuwe bufavirus]* | *51.20%* | *1.09E-05* | *BAR72999* | *40* | *Parvoviridae* | *mammal* |
| *Notamacropus eugenii* | *Tammar wallaby* | *SRR5074479* | *G* |  | *VP1, partial [Simian bufavirus]* | *42.60%* | *2.13E-09* | *APC23175* | *65* | *Parvoviridae* | *mammal* |
| *Notamacropus eugenii* | *Tammar wallaby* | *SRR5074479* | *G* |  | *capsid protein, partial [Cutavirus]* | *53.10%* | *1.14E-06* | *QCH00755* | *48* | *Parvoviridae* | *mammal* |
| *Notamacropus eugenii* | *Tammar wallaby* | *SRR5074479* | *G* |  | *VP1 [Sea otter parvovirus 1]* | *46.20%* | *7.10E-10* | *YP_009272691* | *51* | *Parvoviridae* | *mammal* |
| *Notamacropus eugenii* | *Tammar wallaby* | *SRR5074479* | *G* |  | *VP2 [Gray fox amdovirus]* | *47.10%* | *0.000521* | *YP_009507345* | *46* | *Parvoviridae* | *mammal* |
| *Notamacropus eugenii* | *Tammar wallaby* | *SRR5074478* | *G* |  | *VP1, partial [Bat parvovirus]* | *49.50%* | *7.98E-17* | *AIF74202* | *94* | *Parvoviridae* | *mammal* |
| *Notamacropus eugenii* | *Tammar wallaby* | *SRR5074478* | *G* |  | *VP1 [Sea otter parvovirus 1]* | *57.10%* | *9.46E-07* | *YP_009272691* | *41* | *Parvoviridae* | *mammal* |
| *Notamacropus eugenii* | *Tammar wallaby* | *SRR5074478* | *G* |  | *non-structural protein 3 [Porcine parvovirus]* | *39.70%* | *0.0008* | *ADO22681* | *72* | *Parvoviridae* | *mammal* |
| *Notamacropus eugenii* | *Tammar wallaby* | *SRR5074478* | *G* |  | *VP1 [Rat bufavirus SY-2015]* | *51.30%* | *0.000127* | *YP_009186841* | *38* | *Parvoviridae* | *mammal* |
| *Notamacropus eugenii* | *Tammar wallaby* | *SRR5074478* | *G* |  | *nonstructural protein NS [Goose parvovirus]* | *52.80%* | *2.11E-08* | *AAL37721* | *52* | *Parvoviridae* | *bird* |
| *Notamacropus eugenii* | *Tammar wallaby* | *SRR5074478* | *G* |  | *VP1, partial [Parvovirus fur seal/ATROP46/BR/2012]* | *45.70%* | *5.64E-17* | *AKI82159* | *92* | *Parvoviridae* | *mammal* |
| *Notamacropus eugenii* | *Tammar wallaby* | *SRR5074478* | *G* |  | *VP1 [Sea otter parvovirus 1]* | *52.30%* | *6.99E-16* | *YP_009272691* | *85* | *Parvoviridae* | *mammal* |
| *Notamacropus eugenii* | *Tammar wallaby* | *SRR1552212* | *G* |  | *NS1 [Bufavirus-1]* | *35.70%* | *7.42E-09* | *AFN44270* | *112* | *Parvoviridae* | *mammal* |
| *Notamacropus eugenii* | *Tammar wallaby* | *SRR1552212* | *G* |  | *ZsG-P2A-NP fusion protein [Marburg marburgvirus]* | *65.00%* | *1.00E-38* | *QDH12847* | *19* | *Filoviridae* | *mammal* |
| *Notamacropus eugenii* | *Tammar wallaby* | *SRR1552212* | *G* |  | *viral polymerase [Parrot bornavirus 1]* | *38.00%* | *1.19E-07* | *AFN70793* | *49* | *Bornaviridae* | *bird* |
| *Notamacropus eugenii* | *Tammar wallaby* | *SRR1552212* | *G* |  | *nucleoprotein [Marburg virus - Musoke, Kenya, 1980]* | *39.70%* | *6.07E-06* | *CAA78114* | *59* | *Filoviridae* | *mammal* |
| *Notamacropus eugenii* | *Tammar wallaby* | *SRR1552212* | *G* |  | *nucleoprotein, partial [Zaire ebolavirus]* | *34.10%* | *2.62E-06* | *ARG43197* | *40* | *Filoviridae* | *mammal* |
| *Notamacropus eugenii* | *Tammar wallaby* | *SRR831709* | *G* |  | *nucleoprotein [Bombali ebolavirus]* | *37.80%* | *0.000232* | *QAT98503* | *36* | *Filoviridae* | *mammal* |
| *Notamacropus eugenii* | *Tammar wallaby* | *SRR831709* | *G* |  | *nucleoprotein [Sudan ebolavirus]* | *44.40%* | *3.52E-06* | *AKB09533* | *115* | *Filoviridae* | *mammal* |
| *Notamacropus eugenii* | *Tammar wallaby* | *SRR831709* | *G* |  | *nucleoprotein, partial [Reston ebolavirus]* | *55.00%* | *4.85E-06* | *AUD54831* | *39* | *Filoviridae* | *mammal* |
| *Petrogale xanthopus* | *Yellow footed rock wallaby* | *SRR3901717* | *E* | *33* | *NP [Reston ebolavirus]* | *44.70%* | *2.03E-29* | *ARU80328* | *46* | *Filoviridae* | *mammal* |
| *Petrogale xanthopus* | *Yellow footed rock wallaby* | *SRR3901717* | *E* | *9* | *matrix protein [Loveridges garter snake virus 1]* | *38.90%* | *5.27E-06* | *YP_009055061* | *70* | *Bornaviridae* | *reptile* |
| *Petrogale xanthopus* | *Yellow footed rock wallaby* | *SRR3901717* | *E* | *55* | *ZsG-P2A-NP fusion protein [Marburg marburgvirus]* | *38.10%* | *1.19E-12* | *QDH12847* | *59* | *Filoviridae* | *mammal* |
| *Petrogale xanthopus* | *Yellow footed rock wallaby* | *SRR3901717* | *E* | *6* | *L protein [Wuhan sharpbelly bornavirus]* | *38.10%* | *1.44E-12* | *AVM87541* | *110* | *Bornaviridae* | *fish* |
| *Petrogale xanthopus* | *Yellow footed rock wallaby* | *SRR3901717* | *sc, E* | *12* | *L polymerase [Loveridges garter snake virus 1]* | *29.70%* | *0.000346* | *YP_009055063* | *174* | *Bornaviridae* | *reptile* |
| *Petrogale xanthopus* | *Yellow footed rock wallaby* | *SRR3901717* | *id, E* | *8* | *L polymerase [Loveridges garter snake virus 1]* | *53.10%* | *3.99E-22* | *YP_009055063* | *94* | *Bornaviridae* | *reptile* |
| *Petrogale xanthopus* | *Yellow footed rock wallaby* | *SRR3901717* | *E* | *7* | *L protein [Parrot bornavirus 4]* | *39.70%* | *4.36E-11* | *AEW69865* | *67* | *Bornaviridae* | *bird* |
| *Petrogale xanthopus* | *Yellow footed rock wallaby* | *SRR3901717* | *sh, sc, E* | *8* | *ZsG-P2A-NP fusion protein [Marburg marburgvirus]* | *76.90%* | *1.87E-06* | *QDH12847* | *25* | *Filoviridae* | *mammal* |
| *Petrogale xanthopus* | *Yellow footed rock wallaby* | *SRR3901717* | *sh, E* | *12* | *N [Sudan virus - Boniface, Sudan,1976]* | *47.70%* | *5.92E-18* | *Q9QP77* | *64* | *Filoviridae* | *mammal* |
| *Petrogale xanthopus* | *Yellow footed rock wallaby* | *SRR3901717* | *E* | *42* | *major nucleoprotein [Sudan ebolavirus]* | *33.80%* | *1.07E-10* | *ABY75321* | *149* | *Filoviridae* | *mammal* |
| *Vombatus ursinus* | *Bare nosed wombat* | *ERR2716215* | *G* |  | *glycoprotein [Wuhan sharpbelly bornavirus]* | *39.70%* | *3.17E-16* | *AVM87539* | *125* | *Bornaviridae* | *fish* |
| *Vombatus ursinus* | *Bare nosed wombat* | *ERR2716215* | *G* |  | *Ebolavirus nucleoprotein* | *34.00%* | *2.82E-44* | *5Z9W_A* | *334* | *Filoviridae* | *mammal* |
| *Vombatus ursinus* | *Bare nosed wombat* | *ERR2716215* | *G* |  | *Nucleoprotein [Lake Victoria marburgvirus - Ozolin]* | *41.30%* | *1.52E-35* | *5XSQ_A* | *206* | *Filoviridae* | *mammal* |
| *Vombatus ursinus* | *Bare nosed wombat* | *ERR2716215* | *G* |  | *L polymerase [Loveridges garter snake virus 1]* | *49.30%* | *1.49E-63* | *YP_009055063* | *74* | *Bornaviridae* | *reptile* |
| *Vombatus ursinus* | *Bare nosed wombat* | *ERR2716215* | *G* |  | *L polymerase [Loveridges garter snake virus 1]* | *58.80%* | *1.54E-25* | *YP_009055063* | *79* | *Bornaviridae* | *reptile* |
| *Vombatus ursinus* | *Bare nosed wombat* | *ERR2716215* | *G* |  | *NP, partial [Zaire ebolavirus]* | *57.80%* | *1.60E-39* | *AJB84693* | *108* | *Filoviridae* | *mammal* |
| *Vombatus ursinus* | *Bare nosed wombat* | *ERR2716213* | *sc* |  | *putative nucleoprotein [Guangdong red-banded snake chuvirus-like virus]* | *44.20%* | *2.22E-09* | *AVM87274* | *74* | *RNA* | *reptile* |
| *Vombatus ursinus* | *Bare nosed wombat* | *ERR2716213* | *G* |  | *RNA-dependent RNA polymerase [Borna disease virus 2]* | *51.60%* | *6.12E-20* | *YP_009268922* | *93* | *Bornaviridae* | *mammal* |
| *Vombatus ursinus* | *Bare nosed wombat* | *ERR2716213* | *G* |  | *nucleoprotein [Bundibugyo ebolavirus]* | *38.30%* | *1.08E-73* | *AGL73450* | *182* | *Filoviridae* | *mammal* |
| *Vombatus ursinus* | *Bare nosed wombat* | *ERR2716213* | *G* |  | *glycoprotein [Wuhan sharpbelly bornavirus]* | *39.70%* | *3.17E-16* | *AVM87539* | *125* | *Bornaviridae* | *fish* |
| *Vombatus ursinus* | *Bare nosed wombat* | *ERR2716213* | *G* |  | *Ebolavirus nucleoprotein* | *34.00%* | *4.68E-45* | *5Z9W_A* | *334* | *Filoviridae* | *mammal* |
| *Vombatus ursinus* | *Bare nosed wombat* | *ERR2716213* | *G* |  | *Nucleoprotein [Lake Victoria marburgvirus - Ozolin]* | *41.30%* | *1.52E-35* | *5XSQ_A* | *206* | *Filoviridae* | *mammal* |
| *Vombatus ursinus* | *Bare nosed wombat* | *ERR2716213* | *G* |  | *L polymerase [Loveridges garter snake virus 1]* | *58.80%* | *4.49E-102* | *YP_009055063* | *79* | *Bornaviridae* | *reptile* |
| *Vombatus ursinus* | *Bare nosed wombat* | *ERR2716213* | *G* |  | *VP35 [Zaire ebolavirus]* | *37.20%* | *3.33E-12* | *AKC36417* | *86* | *Filoviridae* | *mammal* |
| *Vombatus ursinus* | *Bare nosed wombat* | *ERR2716211* | *G* |  | *L polymerase [Loveridges garter snake virus 1]* | *58.80%* | *4.49E-102* | *YP_009055063* | *79* | *Bornaviridae* | *reptile* |
| *Vombatus ursinus* | *Bare nosed wombat* | *ERR2716211* | *G* |  | *orfV [Borna disease virus 1]* | *52.60%* | *8.60E-10* | *AAA20667* | *56* | *Bornaviridae* | *mammal* |
| *Vombatus ursinus* | *Bare nosed wombat* | *ERR2716211* | *G* |  | *nucleoprotein [Sudan ebolavirus]* | *39.90%* | *1.16E-74* | *AGL50925* | *182* | *Filoviridae* | *mammal* |
| *Vombatus ursinus* | *Bare nosed wombat* | *ERR2716211* | *G* |  | *Nucleoprotein [Lake Victoria marburgvirus - Ozolin]* | *41.30%* | *1.52E-35* | *5XSQ_A* | *206* | *Filoviridae* | *mammal* |
| *Vombatus ursinus* | *Bare nosed wombat* | *ERR2716211* | *G* |  | *Ebolavirus nucleoprotein* | *34.00%* | *4.68E-45* | *5Z9W_A* | *334* | *Filoviridae* | *mammal* |
| *Vombatus ursinus* | *Bare nosed wombat* | *ERR2716211* | *G* |  | *glycoprotein [Canary bornavirus 1]* | *34.40%* | *7.91E-06* | *YP_009268909* | *91* | *Bornaviridae* | *bird* |
| *Vombatus ursinus* | *Bare nosed wombat* | *ERR2716211* | *G* |  | *polymerase complex protein [Zaire ebolavirus]* | *42.60%* | *0.000429* | *ALR82676* | *51* | *Filoviridae* | *mammal* |
| *Vombatus ursinus* | *Bare nosed wombat* | *ERR2716209* | *G* |  | *VP35 [Zaire ebolavirus]* | *35.20%* | *2.03E-05* | *AKC36417* | *80* | *Filoviridae* | *mammal* |
| *Vombatus ursinus* | *Bare nosed wombat* | *ERR2716209* | *G* |  | *RNA-dependent RNA polymerase [Borna disease virus 2]* | *51.60%* | *6.12E-20* | *YP_009268922* | *93* | *Bornaviridae* | *mammal* |
| *Vombatus ursinus* | *Bare nosed wombat* | *ERR2716209* | *G* |  | *Nucleoprotein [Lake Victoria marburgvirus - Ozolin]* | *41.30%* | *1.52E-35* | *5XSQ_A* | *206* | *Filoviridae* | *mammal* |
| *Vombatus ursinus* | *Bare nosed wombat* | *ERR2716209* | *G* |  | *L polymerase [Loveridges garter snake virus 1]* | *58.80%* | *4.47E-102* | *YP_009055063* | *79* | *Bornaviridae* | *reptile* |
| *Vombatus ursinus* | *Bare nosed wombat* | *ERR2716209* | *G* |  | *Ebolavirus nucleoprotein* | *34.00%* | *2.82E-44* | *5Z9W_A* | *334* | *Filoviridae* | *mammal* |
| *Vombatus ursinus* | *Bare nosed wombat* | *ERR2716209* | *G* |  | *glycoprotein [Estrildid finch bornavirus 1]* | *33.00%* | *1.36E-06* | *YP_009505427* | *100* | *Bornaviridae* | *bird* |
| *Vombatus ursinus* | *Bare nosed wombat* | *ERR2716209* | *G* |  | *nucleoprotein [Bundibugyo ebolavirus]* | *38.30%* | *1.09E-73* | *AGL73450* | *182* | *Filoviridae* | *mammal* |
| *Vombatus ursinus* | *Bare nosed wombat* | *ERR2716209* | *N/A* |  | *hypothetical protein [Sanxia atyid shrimp virus 4]* | *45.50%* | *0.000541* | *YP_009337430* | *43* | *RNA* | *invertebrate* |

**EVE criteria abbreviations: G = genome, id = identity to EVEs, sh = short sequence, sc = stop codons, E = frequently endogenised virus, N/A = not considered an EVE*

*Supplementary Table S3: Actively transcribed endogenous viral elements identified from marsupial RNA-Seq data*

| \| **Animal** \| **Name** \| **Alternate name** \| **Start** \| **End** \| **Sequence Length (NT)** \| **Proximal gene** \| **EVE type** \| **EVE name** \| \| --- \| --- \| --- \| --- \| --- \| --- \| --- \| --- \| --- \| \| Koala \| NW_018344235 \| MSTS010000284.1 \| 1361999 \| 1362619 \| 620 \| TPD52 gene \| *Parvoviridae* \| pcEPLVP1 \| \| Koala \| NW_018344219 \| MSTS01000268.1 \| 1453970 \| 1454914 \| 944 \| EIF4EBP2 gene \| *Filoviridae* \| pcEFLNP1 \| \| Koala \| NW_018344168 \| MSTS01000217.1 \| 884031 \| 884952 \| 921 \| DSE gene \| *Parvoviridae* \| pcEPLVP2 \| \| Koala \| NW_018344082 \| MSTS01000131.1 \| 1317737 \| 1320787 \| 3050 \| FBXL18 gene \| *Bornaviridae* \| pcEBLL1 \| \| Koala \| NW_018344080 \| MSTS01000129.1 \| 6916372 \| 6916602 \| 230 \| - \| *Chuviridae* \| pcECLNP1 \| \| Koala \| NW_018344069 \| MSTS01000118.1 \| 2345507 \| 2346067 \| 560 \| - \| *Bornaviridae* \| pcEBLL2 \| \| Koala \| NW_018344061 \| MSTS01000110.1 \| 1349468 \| 1351427 \| 1959 \| VPS8 gene \| *Parvoviridae* \| pcEPLVP3 \| \| Koala \| NW_018344055 \| MSTS01000104.1 \| 9071112 \| 9071252 \| 140 \| - \| *Bornaviridae* \| pcEBLL3 \| \| Koala \| NW_018344016 \| MSTS01000065.1 \| 12729725 \| 12729913 \| 188 \| SNAP29 gene \| *Chuviridae* \| pcECLNP2 \| \| Koala \| NW_018344014 \| MSTS01000063.1 \| 2637669 \| 2637824 \| 155 \| - \| *Filoviridae* \| pcEFLNP2 \| \| Koala \| NW_018343956 \| MSTS01000005.1 \| 32968313 \| 32969389 \| 1076 \| GABPB2 gene \| *Filoviridae* \| pcEFLNP3 \| \| Koala \| NW_018343953 \| MSTS01000002.1 \| 31890344 \| 31898121 \| 7777 \| NANP gene \| *Filoviridae* \| pcEFLNP4 \| \| Bare-nosed wombat \| NW_020949997 \| UNPS02010219.1 \| 9368192 \| 9374054 \| 5862 \| N/A \| *Filoviridae* \| vuEFLNP1 \| \| Bare-nosed wombat \| NW_020954546 \| UNPS02014768.1 \| 22142127 \| 22143725 \| 1598 \| N/A \| *Bornaviridae* \| vuEBLL1 \| \| Bare-nosed wombat \| NW_020954585 \| UNPS02014807.1 \| 46015686 \| 46016072 \| 386 \| N/A \| *Bornaviridae* \| vuEBLL2 \| \| Bare-nosed wombat \| NW_020954625 \| UNPS02014847.1 \| 4301527 \| 4302267 \| 740 \| N/A \| *Filoviridae* \| vuEFLNP2 \| \| Bare-nosed wombat \| NW_020954737 \| UNPS02014959.1 \| 8427256 \| 8427630 \| 374 \| N/A \| *Bornaviridae* \| vuEBLG1 \| \| Bare-nosed wombat \| NW_020955189 \| UNPS02015411.1 \| 1168084 \| 1168326 \| 242 \| N/A \| ECLNP \| vuECLNP1 \| \| Tasmanian Devil \| NC_045426 \|  \| 200131122 \| 200131400 \| 278 \| LOC100935020 gene \| *Bornaviridae* \| shEBLL1 \| \| Tasmanian Devil \| NC_045426 \|  \| 256006083 \| 256006397 \| 314 \| FBXL18 gene \| *Bornaviridae* \| shEBLL2 \| \| Tasmanian Devil \| NC_045427 \|  \| 388125167 \| 388126523 \| 1356 \| - \| *Parvoviridae* \| shEPLVP1 \| \| Tasmanian Devil \| NC_045428 \|  \| 416517805 \| 416518041 \| 236 \| WDSUB1 gene \| *Parvoviridae* \| shEPLVP2 \| \| Tasmanian Devil \| NC_045429 \|  \| 439443208 \| 439444331 \| 1123 \| LOC116423465 gene/METTL16 gene \| *Bornaviridae* \| shEBLL3 \| \| Tasmanian Devil \| NC_045430 \|  \| 222845874 \| 222846140 \| 266 \| CCDC7 gene \| *Bornaviridae* \| shEBLL4 \| \| Tasmanian Devil \| NC_045431 \|  \| 65535781 \| 65536712 \| 931 \| ELMOD2 gene \| *Bornaviridae* \| shEBLN1 \| \| Tammar wallaby \| Scaffold53193 \|  \| 11726 \| 13603 \| 1877 \|  \| *Parvoviridae* \| meEPLVP1 \| \| Tammar wallaby \| Scaffold99538 \|  \| 5817 \| 6463 \| 646 \|  \| *Filoviridae* \| meEFLNP1 \| \| Tammar wallaby \| Scaffold15426 \|  \| 14825 \| 17334 \| 2509 \|  \| *Parvoviridae* \| meEPLVP2 \| \| Tammar wallaby \| Scaffold19204 \|  \| 8810 \| 8918 \| 108 \|  \| *Parvoviridae* \| meEPLVP3 \| \| Tammar wallaby \| Scaffold385630 \|  \| 459 \| 692 \| 233 \|  \| *Parvoviridae* \| meEPLVP4 \| \| Tammar wallaby \| Scaffold60746 \|  \| 11458 \| 11730 \| 272 \|  \| *Parvoviridae* \| meEPLVP5 \| \| Tammar wallaby \| Scaffold19849 \|  \| 5441 \| 7238 \| 1797 \|  \| *Parvoviridae* \| meEPLVP6 \| \| Tammar wallaby \| Scaffold146633 \|  \| 2112 \| 2322 \| 210 \|  \| *Parvoviridae* \| meEPLVP7 \| \| Tammar wallaby \| GeneScaffold_5926 \|  \| 58164 \| 58301 \| 137 \| ME3 \| *Parvoviridae* \| meEPLVP8 \| \| Tammar wallaby \| GeneScaffold_8425 \|  \| 1 \| 365 \| 364 \|  \| *Filoviridae* \| meEFLNP2 \| \| Tammar wallaby \| GeneScaffold_790 \|  \| 271651 \| 271945 \| 294 \|  \| *Bornaviridae* \| meEBLL1 \| \| Tammar wallaby \| Scaffold155090 \|  \| 6607 \| 6944 \| 337 \|  \| *Filoviridae* \| meEFLNP3 \| \| Tammar wallaby \| GeneScaffold_5731 \|  \| 22688 \| 23474 \| 786 \|  \| *Filoviridae* \| meEFLNP4 \| \| Brushtail possum \| CM021909 \|  \| 108336286 \| 108338478 \| 2192 \|  \| *Filoviridae* \| tvEFLNP1 \| |
| --- | --- | --- | --- | --- | --- | --- | --- | --- | --- | --- | --- | --- | --- | --- | --- | --- | --- | --- | --- | --- | --- | --- | --- | --- | --- | --- | --- | --- | --- | --- | --- | --- | --- | --- | --- | --- | --- | --- | --- | --- | --- | --- | --- | --- | --- | --- | --- | --- | --- | --- | --- | --- | --- | --- | --- | --- | --- | --- | --- | --- | --- | --- | --- | --- | --- | --- | --- | --- | --- | --- | --- | --- | --- | --- | --- | --- | --- | --- | --- | --- | --- | --- | --- | --- | --- | --- | --- | --- | --- | --- | --- | --- | --- | --- | --- | --- | --- | --- | --- | --- | --- | --- | --- | --- | --- | --- | --- | --- | --- | --- | --- | --- | --- | --- | --- | --- | --- | --- | --- | --- | --- | --- | --- | --- | --- | --- | --- | --- | --- | --- | --- | --- | --- | --- | --- | --- | --- | --- | --- | --- | --- | --- | --- | --- | --- | --- | --- | --- | --- | --- | --- | --- | --- | --- | --- | --- | --- | --- | --- | --- | --- | --- | --- | --- | --- | --- | --- | --- | --- | --- | --- | --- | --- | --- | --- | --- | --- | --- | --- | --- | --- | --- | --- | --- | --- | --- | --- | --- | --- | --- | --- | --- | --- | --- | --- | --- | --- | --- | --- | --- | --- | --- | --- | --- | --- | --- | --- | --- | --- | --- | --- | --- | --- | --- | --- | --- | --- | --- | --- | --- | --- | --- | --- | --- | --- | --- | --- | --- | --- | --- | --- | --- | --- | --- | --- | --- | --- | --- | --- | --- | --- | --- | --- | --- | --- | --- | --- | --- | --- | --- | --- | --- | --- | --- | --- | --- | --- | --- | --- | --- | --- | --- | --- | --- | --- | --- | --- | --- | --- | --- | --- | --- | --- | --- | --- | --- | --- | --- | --- | --- | --- | --- | --- | --- | --- | --- | --- | --- | --- | --- | --- | --- | --- | --- | --- | --- | --- | --- | --- | --- | --- | --- | --- | --- | --- | --- | --- | --- | --- | --- | --- | --- | --- | --- | --- | --- | --- | --- | --- | --- | --- | --- | --- | --- | --- | --- | --- | --- | --- | --- | --- | --- | --- | --- | --- | --- | --- | --- | --- | --- | --- | --- | --- | --- | --- | --- | --- | --- | --- | --- | --- | --- | --- | --- | --- | --- | --- | --- | --- | --- |

*^a^ NP = nucleoprotein, L = replicase, G = glycoprotein, M = matrix protein, VP = viral protein (capsid), NS = non-structural protein*

*Supplementary Table S4 : Small RNA datasets used in this study*

| \| **Species** \| **Tissue** \| **Accession** \| **Spots** \| \| --- \| --- \| --- \| --- \| \| Tasmanian devil \| DFTD3 \| SRR034103 \| 1,520,338 \| \| Tasmanian devil \| Kidney \| SRR034104 \| 1,650,778 \| \| Tasmanian devil \| Brain \| SRR034105 \| 1,988,662 \| \| Tasmanian devil \| Heart \| SRR034106 \| 1,309,876 \| \| Tasmanian devil \| Liver \| SRR034107 \| 1,688,979 \| \| Tasmanian devil \| Bone marrow \| SRR034108 \| 1,174,570 \| \| Tasmanian devil \| Pancreas \| SRR034109 \| 443,441 \| \| Tasmanian devil \| Skin \| SRR034110 \| 1,327,708 \| \| Tasmanian devil \| Spleen \| SRR034111 \| 1,569,800 \| \| Tasmanian devil \| DFTD2 \| SRR034112 \| 35,170 \| \| Tasmanian devil \| DFTD1 \| SRR034113 \| 2,588,174 \| \| Tasmanian devil \| DFTD2 \| SRR034114 \| 2,542,936 \| \| Tasmanian devil \| DFTD20 \| SRR034115 \| 2,420,961 \| \| Tasmanian devil \| Testis \| SRR034116 \| 2,273,621 \| \| Tasmanian devil \| Lymph node \| SRR034117 \| 429,566 \| \| Koala \| Testis \| SRR8708142 \| 38,186,977 \| \| Koala \| Testis \| SRR8708143 \| 42,786,287 \| \| Koala \| Liver \| SRR8708144 \| 38,629,504 \| \| Koala \| Brain \| SRR8708145 \| 44,516,823 \| \| Koala \| Testis \| SRR8708146 \| 46,740,366 \| \| Koala \| Testis \| SRR8708147 \| 54,188,217 \| \| Koala \| Liver \| SRR8708148 \| 36,289,742 \| \| Koala \| Brain \| SRR8708149 \| 40,370,112 \| \| Koala \| Testis \| SRR8708150 \| 51,051,104 \| \| Koala \| Testis \| SRR8708151 \| 48,794,299 \| \| Tammar wallaby \| Brain \| SRR306310 \| 161,246 \| \| Tammar wallaby \| Fibroblast \| SRR306311 \| 4,879,429 \| \| Tammar wallaby \| Liver \| SRR306312 \| 404,177 \| \| Tammar wallaby \| Milk \| SRR1501606 \| 10,205,531 \| \| Tammar wallaby \| Milk \| SRR1501605 \| 14,309,026 \| \| Tammar wallaby \| Milk \| SRR1501604 \| 13,709,829 \| \| Tammar wallaby \| Milk \| SRR1501603 \| 12,309,932 \| \| Tammar wallaby \| Milk \| SRR1501602 \| 13,784,187 \| \| Tammar wallaby \| Milk \| SRR1501601 \| 12,134,643 \| \| Tammar wallaby \| Milk \| SRR1501600 \| 12,381,908 \| |  |  |
| --- | --- | --- | --- | --- | --- | --- | --- | --- | --- | --- | --- | --- | --- | --- | --- | --- | --- | --- | --- | --- | --- | --- | --- | --- | --- | --- | --- | --- | --- | --- | --- | --- | --- | --- | --- | --- | --- | --- | --- | --- | --- | --- | --- | --- | --- | --- | --- | --- | --- | --- | --- | --- | --- | --- | --- | --- | --- | --- | --- | --- | --- | --- | --- | --- | --- | --- | --- | --- | --- | --- | --- | --- | --- | --- | --- | --- | --- | --- | --- | --- | --- | --- | --- | --- | --- | --- | --- | --- | --- | --- | --- | --- | --- | --- | --- | --- | --- | --- | --- | --- | --- | --- | --- | --- | --- | --- | --- | --- | --- | --- | --- | --- | --- | --- | --- | --- | --- | --- | --- | --- | --- | --- | --- | --- | --- | --- | --- | --- | --- | --- | --- | --- | --- | --- | --- | --- | --- | --- | --- | --- | --- | --- | --- | --- | --- | --- |
|  |  |  |
|  |  |  |

*Supplementary Table S5: Small RNA mapped to genomic EVEs and EVE transcripts*

|  |  | Number of mapped small RNA | | % mapped to EVE transcripts | | % mapped to genomic EVEs | |
| --- | --- | --- | --- | --- | --- | --- | --- |
| Tissue | Accession | siRNA | piRNA | siRNA | piRNA | siRNA | piRNA |
| Testis | SRR8708142 | 7288 | 63,738 | 0.01 | 0.05 | 0.11 | 0.41 |
| Testis | SRR8708143 | 5621 | 78573 | 0.01 | 0.06 | 0.11 | 0.42 |
| Liver | SRR8708144 | 464 | 748 | 0.00 | 0.00 | 0 | 0.01 |
| Brain | SRR8708145 | 319 | 459 | 0.00 | 0.00 | 0 | 0 |
| Testis | SRR8708146 | 4213 | 47789 | 0.00 | 0.05 | 0.07 | 0.35 |
| Testis | SRR8708147 | 5617 | 78872 | 0.00 | 0.06 | 0.08 | 0.39 |
| Liver | SRR8708148 | 589 | 935 | 0.00 | 0.00 | 0 | 0 |
| Brain | SRR8708149 | 915 | 1318 | 0.00 | 0.00 | 0.01 | 0.01 |
| Testis | SRR8708150 | 4909 | 158139 | 0.02 | 0.07 | 0.35 | 0.47 |
| Testis | SRR8708151 | 4642 | 140968 | 0.02 | 0.08 | 0.31 | 0.42 |
